# Prediction models for the recipients’ ideal perioperative estimated glomerular filtration rates for predicting graft survival after adult living-donor kidney transplantation

**DOI:** 10.3389/fmed.2023.1187777

**Published:** 2023-08-31

**Authors:** Takahisa Hiramitsu, Yuki Hasegawa, Kenta Futamura, Manabu Okada, Yutaka Matsuoka, Norihiko Goto, Toshihiro Ichimori, Shunji Narumi, Asami Takeda, Takaaki Kobayashi, Kazuharu Uchida, Yoshihiko Watarai

**Affiliations:** ^1^Department of Transplant and Endocrine Surgery, Japanese Red Cross Aichi Medical Center Nagoya Daini Hospital, Nagoya, Japan; ^2^Department of Renal Transplant Surgery, Masuko Memorial Hospital, Nagoya, Japan; ^3^Department of Nephrology, Japanese Red Cross Aichi Medical Center Nagoya Daini Hospital, Nagoya, Japan; ^4^Department of Renal Transplant Surgery, Aichi Medical University School of Medicine, Nagakute, Japan

**Keywords:** cross-validation, estimated glomerular filtration rate, graft survival, living-donor kidney transplantation, prediction model

## Abstract

**Introduction:**

The impact of the perioperative estimated glomerular filtration rate (eGFR) on graft survival in kidney transplant recipients is yet to be evaluated. In this study, we developed prediction models for the ideal perioperative eGFRs in recipients.

**Methods:**

We evaluated the impact of perioperative predicted ideal and actual eGFRs on graft survival by including 1,174 consecutive adult patients who underwent living-donor kidney transplantation (LDKT) between January 2008 and December 2020. Prediction models for the ideal perioperative eGFR were developed for 676 recipients who were randomly assigned to the training and validation sets (ratio: 7:3). The prediction models for the ideal best eGFR within 3 weeks and those at 1, 2, and 3 weeks after LDKT in 474 recipients were developed using 10-fold validation and stepwise multiple regression model analyzes. The developed prediction models were validated in 202 recipients. Finally, the impact of perioperative predicted ideal eGFRs/actual eGFRs on graft survival was investigated using Fine–Gray regression analysis.

**Results:**

The correlation coefficients of the predicted ideal best eGFR within 3 weeks and the predicted ideal eGFRs at 1, 2, and 3 weeks after LDKT were 0.651, 0.600, 0.598, and 0.617, respectively. Multivariate analyzes for graft loss demonstrated significant differences in the predicted ideal best eGFR/actual best eGFR within 3 weeks and the predicted ideal eGFRs/actual eGFRs at 1, 2, and 3 weeks after LDKT.

**Discussion:**

The predicted ideal best eGFR/actual best eGFR within 3 weeks and the predicted ideal eGFRs/actual eGFRs at 1, 2, and 3 weeks after LDKT were independent prognostic factors for graft loss. Therefore, the perioperative predicted ideal eGFR/actual eGFR may be useful for predicting graft survival after adult LDKT.

## 1. Introduction

Donor and recipient characteristics, operative factors, postoperative complications, and immunosuppressive drugs may affect graft function after living-donor kidney transplantation (LDKT). Specifically, donor and recipient characteristics, including donor age, recipient sex, and donor estimated glomerular filtration rate (eGFR), graft pathological features, and anti-human leukocyte antigen (HLA) donor-specific antibodies (DSAs) affect postoperative graft function (1–3). Postoperative graft function is also affected by operative factors and postoperative complications, including laparoscopic nephrectomy, warm ischemia time, urological and vascular complications, and rejection (4–7). Calcineurin inhibitors can cause nephrotoxicity and lead to a low eGFR (8, 9). Furthermore, postoperative graft function is considered a good predictor of graft survival. Studies have investigated the impact of the eGFR on graft survival at 1 year after kidney transplantation (KT) (2, 10–16). However, the effect of the perioperative eGFR on graft survival in LDKT is yet to be investigated. Previously, perioperative graft function was stratified and evaluated based on slow or delayed graft function (17, 18). Several studies have developed prediction models for recipients’ eGFRs at 1–5 years after KT (19–21). However, to our knowledge, no study has reported the development of prediction models for ideal eGFRs during perioperative LDKT. Therefore, we investigated the impact of perioperative actual eGFRs on graft survival in adult LDKT. Additionally, we developed prediction models for recipients’ ideal eGFRs during the perioperative period to investigate the impact of predicted ideal eGFRs on graft survival.

## 2. Materials and methods

### 2.1. Study design

This single-center retrospective cohort study was approved by the Nagoya Daini Red Cross Hospital’s Institutional Review Board (Aichi, Japan; approval number: 1504) and was conducted following the principles of the Declaration of Helsinki. The study included 1,174 consecutive adult patients who underwent LDKT between January 2008 and December 2020. First, the impacts of the actual best eGFR within 3 weeks after LDKT and actual eGFRs at 1, 2, and 3 weeks and at 1, 3, 6, and 12 months after LDKT on graft survival were investigated in 1174 recipients. Second, prediction models were developed for the ideal best eGFR within 3 weeks and ideal eGFRs at 1, 2, and 3 weeks after LDKT. We developed prediction models based on 676 ideal recipients selected from 1,174 recipients. Finally, the impact of the predicted ideal best eGFR/actual best eGFR within 3 weeks and the predicted ideal eGFRs/actual eGFRs at 1, 2, and 3 weeks after LDKT on graft survival was investigated in 1174 recipients. This study was reported following the Strengthening the Reporting of Observational Studies in Epidemiology (STROBE) guidelines.

### 2.2. Participants

**Figure 1:**
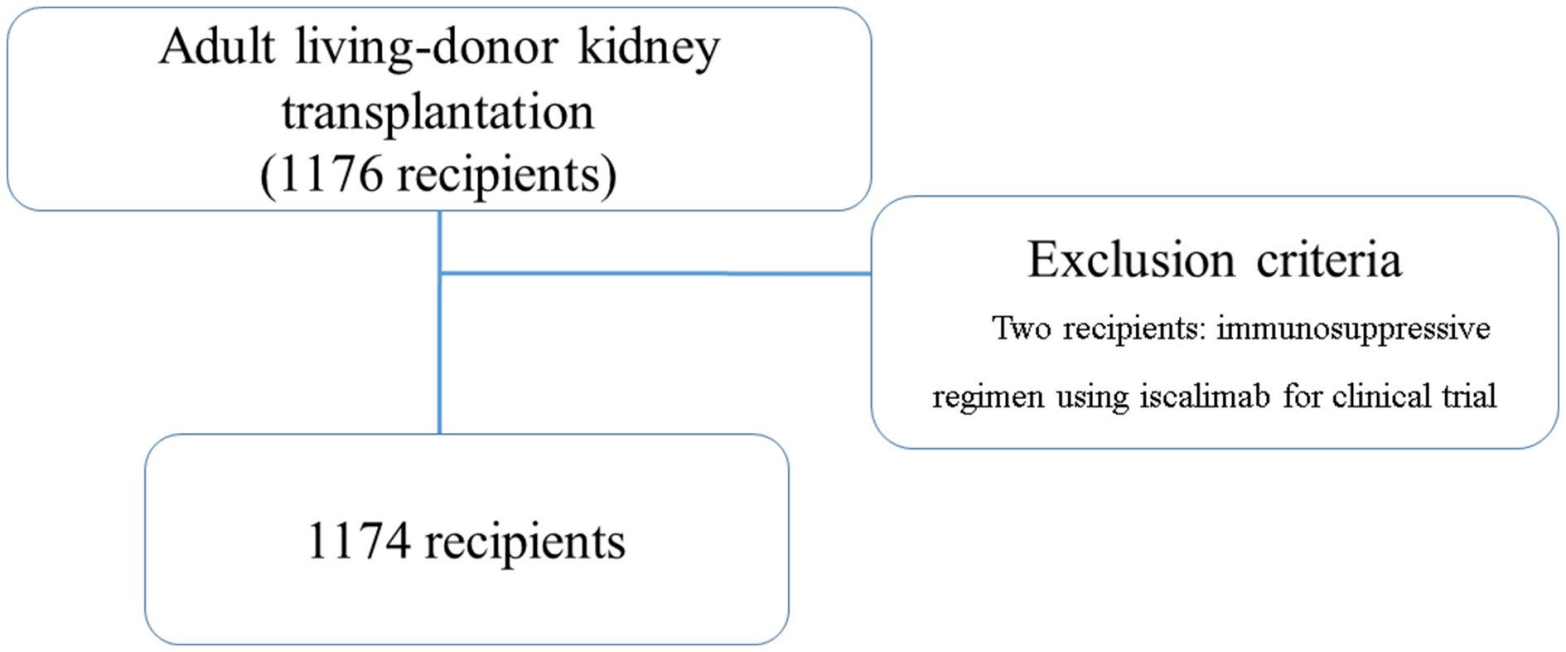
Patient flowchart.

This study included all consecutive recipients who underwent LDKT at our hospital between January 2008 and December 2020. The recipients were followed up until August 2021. Additionally, we excluded recipients with an immunosuppressive regimen using iscalimab in clinical trials (two recipients) (Figure 1). All donor and recipient data were retrospectively collected from the medical records and analyzed anonymously; therefore, the requirement for informed consent was waived by the Nagoya Daini Red Cross Hospital’s Institutional Review Board (Aichi, Japan; approval number: 1504).

### 2.3. Living donors

Living donors were selected according to the guidelines for living kidney donors in Japan (22). The laterality of the kidney for donor nephrectomy was determined using the results of technetium-99 m diethylene triamine pentaacetic acid (Tc-99m DTPA). A difference in Tc-99m DTPA ≥10% between the right and left kidneys indicated a nephrectomy of the inferior side. In contrast, a discrepancy in Tc-99m DTPA of <10% indicated left nephrectomy. Furthermore, data on donor characteristics, surgical outcomes, and perioperative complications were collected and analyzed.

### 2.4. Recipients

LDKTs were performed following the Istanbul Declaration. The recipients stayed at the hospital for 3 weeks after LDKT. After discharge, postoperative recipient assessments were performed fortnightly for the first 3 months and subsequently monthly at our hospital and local hospitals. Protocol biopsies were performed at 1 h after reperfusion as a baseline, and at 1 month after KT.

Data on donor and recipient characteristics and operative outcomes, actual eGFR after LDKT, graft survival, and recipient mortality were collected. These data were used to analyze the impact of the actual best eGFR within 3 weeks after LDKT as well as actual eGFRs at 1, 2, and 3 weeks and at 1, 3, 6, and 12 months after LDKT on graft survival. Additionally, to develop the prediction models for the ideal best eGFR within 3 weeks and ideal eGFRs at 1, 2, and 3 weeks after LDKT, data on donor and recipient characteristics and operative outcomes; perioperative adverse events; best eGFR within 3 weeks after LDKT; and actual eGFRs at 1, 2, and 3 weeks after LDKT were collected and analyzed. Furthermore, prediction models were developed for recipients with ideal graft conditions within 1 month after LDKT. Recipients who received grafts from donors with intraoperative adverse events; those who received transplanted grafts with arterial reconstruction or ligation of the thin upper pole artery; and those who experienced perioperative adverse events, conversion of the immunosuppressive regimen, recurrence of nephritis, calcineurin inhibitor toxicity, and rejection within 1 month were excluded from the development of the prediction models for ideal eGFRs within 3 weeks. The detailed reasons for excluding 498 recipients from the development of the prediction models are presented in Supplementary Table S1. Data on donor and recipient characteristics, predicted ideal eGFR/actual eGFR, graft survival, and recipient mortality were collected to investigate the impact of the predicted ideal eGFR/actual eGFR on graft survival.

### 2.5. Immunosuppressive protocols

For the ABO-compatible KT, basiliximab, steroids, calcineurin inhibitors (i.e., cyclosporin, tacrolimus, or extend-release tacrolimus), and an antimetabolite or mammalian target of rapamycin inhibitor (i.e., mycophenolate mofetil, mizoribine, or everolimus) were administered for induction and maintenance therapy. Desensitization was performed using rituximab or splenectomy, double-filtration plasmapheresis, and plasmapheresis for the ABO-incompatible KT. Basiliximab, steroids, and calcineurin inhibitors (i.e., cyclosporin, tacrolimus or extend-release tacrolimus), and mycophenolate mofetil were administered for induction and maintenance therapy. Regarding the preformed-DSA KT, desensitization was performed using rituximab, double-filtration plasmapheresis, plasmapheresis, or intravenous immunoglobulin administration. Furthermore, basiliximab, steroids, calcineurin inhibitors (i.e., tacrolimus or extend-release tacrolimus), and mycophenolate mofetil were administered for induction and maintenance therapy.

### 2.6. Statistical analysis

**Figure 2:**
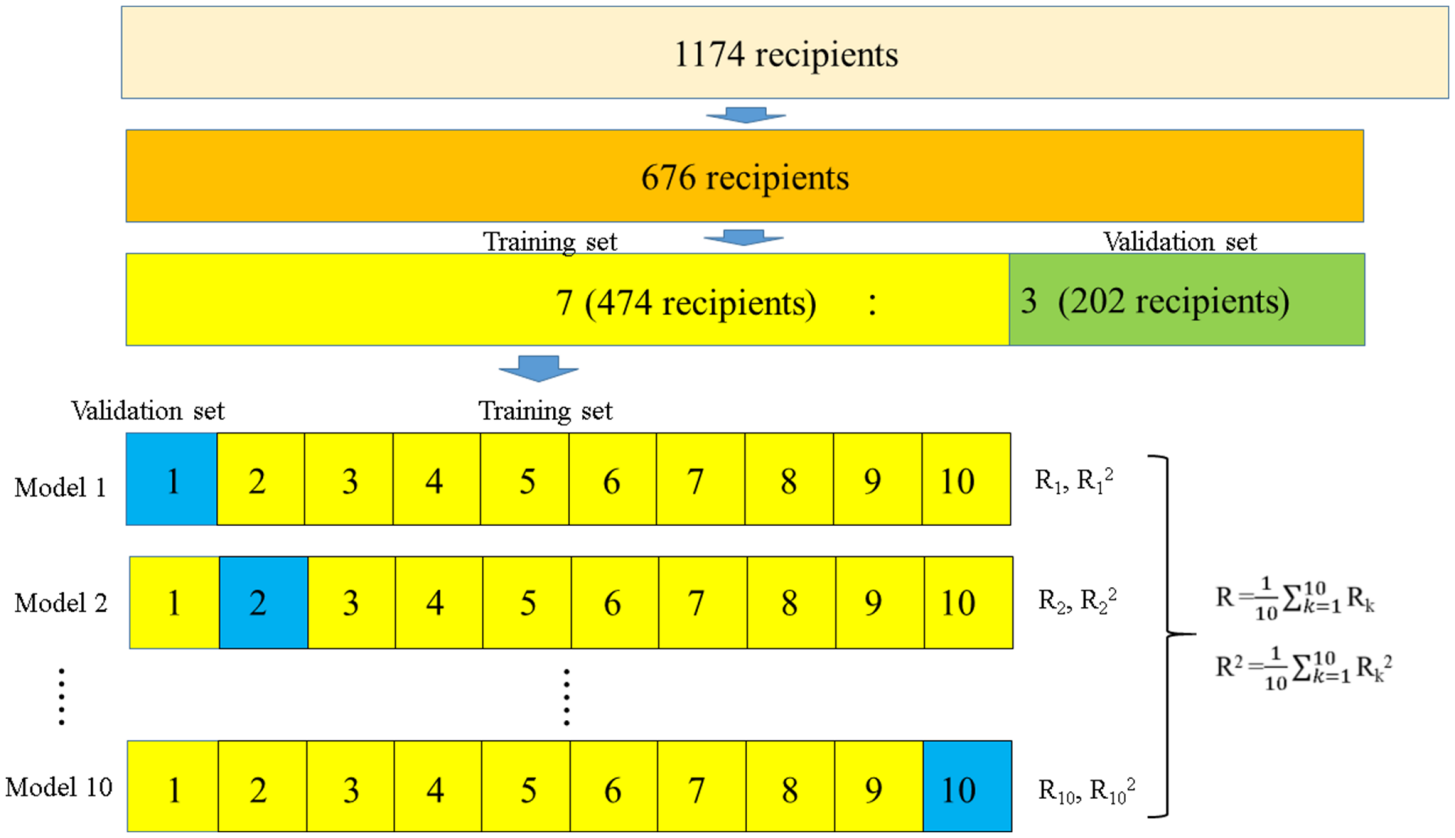
Flowchart of the development of the prediction models for ideal eGFRs using 10-fold validation. Overall, 676 ideal recipients were selected to develop the prediction models. The recipients were randomly categorized into two groups in a 7:3 ratio (474 and 202 recipients for the training and validation sets, respectively). In the 474 recipients, 10-fold validation and stepwise multiple regression model analyzes were used to develop prediction models for ideal eGFRs, while the developed prediction models were validated in 202 patients. eGFR, estimated glomerular filtration rate.

Statistical analyzes of donor and recipient characteristics were performed using the Kruskal–Wallis test and the chi-square or Fisher’s exact test for continuous and categorical variables, respectively. An estimation equation model was constructed to predict the eGFR. The independent variables used in the estimation equation were initially tested for collinearity in advance, and factors with collinearity were excluded to prevent overfitting the model. Subsequently, estimation equations were constructed on the training set and confirmed using the validation set. The patients were randomly categorized into two groups in a 7:3 ratio, of whom 474 and 202 were assigned to the training and validation sets, respectively (Figure 2).

A linear regression prediction model was constructed using the eGFR as the dependent variable of the training set to establish an equation for estimating the eGFR. Subsequently, a stepwise method with 10-fold validation was used to limit the variables to be included in the model, and the estimation accuracy was evaluated. The R and R-squared values were used to estimate the accuracy. Finally, the constructed estimation equations were evaluated for their accuracy on the validation set.

A Fine–Gray competing risk regression model was used to determine the prognostic factors for graft loss. The proportional hazard assumption was confirmed using a log–log plot for the Fine–Gray competing risk regression model. No interaction effects between the variables were found in the models using the interaction items. Covariates with a *p*-value <0.05 in the univariate logistic regression analysis were used in the multivariate logistic regression analysis. Statistical significance was set at 0.05 (two-sided). All analyzes were performed using the Statistical Package for the Social Sciences (Version 24.0, IBM Japan Ltd., Tokyo, Japan) and R version 4.0.3 (R Core Team [2020], Vienna, Austria).

## 3. Results

### 3.1. Study population

Overall, 1,176 adult LDKTs were performed at our hospital during this study, of which two LDKTs were excluded, and the remaining 1,174 recipients were included. The 1,174 recipients were followed up between January 2008 and August 2021 (median observation period: 77.0 [interquartile range, 45.0–117.0] months) and were included in the final analysis.

### 3.2. Recipient results

#### 3.2.1. Descriptive data concerning donors and recipients

The characteristics of donors and recipients are presented in Table 1. Using the Fine–Gray competing risk regression model, recipients were presented in the following three groups: recipients with functioning grafts (1,059 patients), graft loss (73 patients), and death with functioning grafts (42 patients). Regarding donor characteristics, significant differences were observed in donor age (*p* = 0.002); donation to first-degree relative recipients (*p* = 0.001); preoperative comorbidities ≥1 (hypertension: blood pressure > 140/90 mmHg or treatment with blood pressure-lowering medications; dyslipidemia: low-density lipoprotein cholesterol level > 140 mg/dL, triglyceride level > 150 mg/dL, high-density lipoprotein cholesterol level < 40 mg/dL, or treatment of dyslipidemia; glucose intolerance: impaired fasting glycemia, impaired glucose tolerance, or diabetes mellitus without insulin treatment; and obesity: body mass index [BMI] >30 kg/m^2^) (*p* = 0.015); preoperative systolic blood pressure (*p* = 0.003); preoperative diastolic blood pressure (*p* = 0.023); preoperative hemoglobin A1c level (*p* = 0.039); preoperative BMI (*p* = 0.027); preoperative urine albumin/creatine ratio (*p* = 0.014); and baseline biopsy findings at 1 h after transplantation (presence of interstitial fibrosis, tubular atrophy, arteriolosclerosis, or glomerulosclerosis based on the 2018 Banff classification) (*p* = 0.048) (23).

**Table 1:** Donor and recipient characteristics.

		Functioning graft	Graft loss	Death with functioning grafts	*p-*value
		*n* = 1,059	*n* = 73	*n* = 42	
Donor					
Donor age (years, SD)		58.6 (10.0)	60.3 (10.0)	63.4 (8.2)	**0.002**
Donor sex (male, %)		390 (36.8)	27 (37.0)	14 (33.3)	0.898
Donation to first-degree relative recipients (%)		487 (46.0)	43 (58.9)	10 (23.8)	**0.001**
Smoking history (%)		474 (44.8)	34 (46.6)	14 (33.3)	0.320
Preoperative comorbidities ≥1 (%)		764 (72.1)	60 (82.2)	37 (88.1)	**0.015**
	Hypertension (%)	300 (28.3)	34 (46.6)	22 (52.4)	**<0.001**
	Dyslipidemia (%)	610 (57.6)	46 (63.0)	32 (76.2)	**0.041**
	Glucose intolerance (%)	284 (26.8)	27 (37.0)	13 (31.0)	0.151
	Obesity—body mass index ≥30 kg/m^2^ (%)	5 (0.5)	2 (2.7)	0	**0.045**
Donor preoperative systolic blood pressure (mmHg, SD)		122.8 (14.5)	127.3 (13.3)	127.0 (11.8)	**0.003**
Donor preoperative diastolic blood pressure (mmHg, SD)		73.7 (10.8)	75.9 (10.3)	76.7 (11.3)	**0.023**
Donor preoperative total cholesterol level (mg/dL, SD)		211.6 (37.0)	206.0 (32.6)	217.2 (33.7)	0.264
Donor preoperative triglyceride level (mg/dL, SD)		139.3 (87.1)	137.2 (79.5)	152.1 (90.4)	0.581
Donor preoperative low-density lipoprotein cholesterol level (mg/dL, SD)		123.3 (30.8)	122.9 (28.8)	126.6 (29.2)	0.704
Donor preoperative high-density lipoprotein cholesterol level (mg/dL, SD)		63.0 (16.3)	50.3 (15.9)	64.3 (24.4)	0.454
Donor preoperative fasting glucose level (mg/dL, SD)		99.3 (12.5)	99.5 (12.8)	97.1 (9.7)	0.597
Donor preoperative 75-g oral glucose tolerance test results—blood glucose level at 2 h after glucose administration (mg/dL, SD)		131.2 (36.3)	139.5 (49.4)	133.6 (34.3)	0.358
Donor preoperative HbA1c level (%, SD)		5.7 (0.4)	5.8 (0.4)	5.8 (0.3)	**0.039**
Donor preoperative body mass index (kg/m^2^, SD)		22.7 (2.8)	23.2 (3.3)	23.8 (2.7)	**0.027**
Donor preoperative eGFR (mL/min/1.73 m^2^, SD)		73.4 (12.7)	73.7 (13.5)	72.6 (18.9)	0.382
Donor preoperative split kidney function on Tc-99m DTPA scintigraphy (%, SD)		48.0 (3.7)	48.3 (3.6)	47.2 (3.3)	0.239
Preoperative urine albumin/Cr ratio (mg/g Cr, SD)		9.4 (11.6)	15.8 (24.4)	11.3 (10.4)	**0.014**
Baseline biopsy findings at 1 h after transplantation (%)		582 (55.4)	37 (51.4)	29 (74.4)	**0.048**
Recipient					
Recipient age (years, SD)		48.6 (13.7)	46.7 (14.9)	60.9 (8.9)	**<0.001**
Recipient sex (male, %)		658 (62.1)	53 (72.6)	28 (66.7)	0.177
Cause of end-stage renal disease	Diabetes mellitus (%)	197 (18.6)	13 (17.8)	15 (35.7)	**0.019**
	Glomerulonephritis (%)	414 (39.1)	31 (42.5)	12 (28.6)	
	Hypertension (%)	78 (7.4)	6 (8.2)	2 (4.8)	
	Polycystic kidney disease (%)	88 (8.3)	0	6 (14.3)	
	Others (%)	282 (26.6)	23 (31.5)	7 (16.7)	
Recipient body mass index (kg/m^2^, SD)		22.4 (3.7)	23.0 (4.4)	22.4 (3.7)	0.515
Recipient follow-up period (months, SD)		74.4 (44.5)	101.8 (37.6)	66.5 (40.1)	**<0.001**
Transplantation from first-degree relative donors (%)		487 (46.0)	43 (58.9)	10 (23.8)	**0.001**
Preoperative flow cytometry T cell crossmatch (positive, %)		37 (3.5)	2 (2.7)	2 (4.8)	0.851
Preoperative flow cytometry B cell crossmatch (positive, %)		94 (8.9)	14 (19.2)	6 (14.3)	**0.010**
Dialysis vintage (months, SD)		73.7 (392.7)	30.8 (46.9)	89.0 (195.0)	**<0.001**
Preoperative ejection fraction on ultrasonographic cardiography (%)		61.9 (7.6)	61.8 (8.7)	53.5 (15.7)	0.580
Preoperative ventricular wall motion asynergy on ultrasonographic cardiography (%)		125 (11.8)	11 (15.3)	10 (23.8)	0.053
Preoperative sensitization—transfusion, pregnancy, transplantation (%)		437 (41.3)	25 (34.2)	18 (42.9)	0.481
HLA-AB mismatch (SD)		2.4 (1.0)	2.3 (0.9)	2.9 (0.9)	**0.010**
HLA-DR mismatch (SD)		1.4 (0.6)	1.3 (0.5)	1.6 (0.6)	0.056
Preoperative PRA class I (positive, ≥5%, %)		153 (14.4)	12 (16.4)	4 (9.5)	0.589
Preoperative PRA class II (positive, ≥5%, %)		86 (8.1)	4 (5.5)	1 (2.4)	0.298
Preformed DSA (%)		70 (6.6)	10 (13.7)	3 (7.1)	0.073
	Preoperative flow cytometry T cell crossmatch after desensitization for preformed DSA (positive, %)	10 (14.3)	0	1 (33.3)	0.292
	Preoperative flow cytometry B cell crossmatch after desensitization for preformed DSA (positive, %)	56 (80.0)	7 (77.8)	2 (66.7)	0.850
	ICFA class I after desensitization for preformed DSA (positive, %)	3 (5.9)	0	0	0.940
	ICFA class II after desensitization for preformed DSA (positive, %)	4 (7.8)	0	0	0.919
ABO-incompatible transplantation (%)		346 (32.7)	28 (38.4)	18 (42.9)	0.253
Preoperative desensitization (preoperative rituximab administration or splenectomy, preoperative double-filtration plasmapheresis, plasmapheresis, or IVIG, %)		393 (37.1)	36 (49.3)	19 (45.2)	0.073
Calcineurin inhibitor administration at kidney transplantation	TAC (%)	193 (18.2)	22 (30.1)	14 (33.3)	**<0.001**
	CsA (%)	370 (34.9)	42 (57.5)	24 (57.1)	
	TACER (%)	496 (46.8)	9 (12.3)	4 (9.5)	
Calcineurin inhibitor administration at best eGFR within 3 weeks after kidney transplantation	TAC (%)	192 (18.1)	21 (28.8)	14 (33.3)	**<0.001**
	CsA (%)	369 (34.8)	42 (57.5)	24 (57.1)	
	TACER (%)	498 (47.0)	10 (13.7)	4 (9.5)	
Calcineurin inhibitor administration at 1 week after kidney transplantation	TAC (%)	191 (18.0)	20 (27.4)	14 (33.3)	**<0.001**
	CsA (%)	373 (35.2)	43 (58.9)	24 (57.1)	
	TACER (%)	495 (46.7)	10 (13.7)	4 (9.5)	
Calcineurin inhibitor administration at 2 weeks after kidney transplantation	TAC (%)	192 (18.1)	21 (28.8)	14 (33.3)	**<0.001**
	CsA (%)	371 (35.1)	43 (58.9)	24 (57.1)	
	TACER (%)	495 (46.8)	9 (12.3)	4 (9.5)	
Calcineurin inhibitor administration at 3 weeks after kidney transplantation	TAC (%)	182 (17.6)	18 (26.5)	13 (32.5)	**<0.001**
	CsA (%)	359 (34.7)	41 (60.3)	23 (57.5)	
	TACER (%)	495 (47.8)	9 (13.2)	4 (10.0)	
MMF, MZ, or EVR administration at transplantation	MMF (%)	842 (79.5)	68 (93.2)	34 (81.0)	**0.049**
	MZ (%)	32 (3.0)	2 (2.7)	2 (4.8)	
	EVR (%)	185 (17.5)	3 (4.1)	6 (14.3)	
Conversion of immunosuppressive regimen within 1 month (%)		13 (1.2)	2 (2.7)	0	0.406
Actual best eGFR within 3 weeks (mL/min/1.73 m^2^, SD)		58.0 (16.2)	55.2 (22.4)	53.5 (15.7)	**0.039**
Actual eGFR at 1 week (mL/min/1.73 m^2^, SD)		50.2 (15.1)	47.7 (21.7)	45.0 (15.4)	**0.038**
Actual eGFR at 2 weeks (mL/min/1.73 m^2^, SD)		49.6 (14.5)	46.9 (21.1)	44.7 (12.1)	**0.013**
Actual eGFR at 3 weeks (mL/min/1.73 m^2^, SD)		48.8 (14.2)	45.5 (20.1)	45.7 (11.1)	0.055
Actual eGFR at 1 month (mL/min/1.73 m^2^, SD)		47.4 (13.1)	44.0 (18.1)	45.9 (15.5)	0.060
Actual eGFR at 3 months (mL/min/1.73 m^2^, SD)		45.2 (12.1)	41.4 (19.1)	42.9 (14.0)	**0.025**
Actual eGFR at 6 months (mL/min/1.73 m^2^, SD)		45.2 (11.5)	38.2 (16.9)	41.7 (11.7)	**<0.001**
Actual eGFR at 12 months (mL/min/1.73 m^2^, SD)		45.0 (11.8)	36.3 (14.7)	41.5 (12.3)	**<0.001**
Trough levels of calcineurin inhibitor	TAC at best eGFR (ng/mL)	11.1 (4.5)	12.1 (7.5)	11.6 (3.9)	0.797
	TAC at 1 week (ng/mL)	11.1 (3.8)	12.6 (4.5)	12.2 (3.7)	0.100
	TAC at 2 weeks (ng/mL)	10.5 (2.9)	9.8 (3.7)	12.0 (3.1)	0.080
	TAC at 3 weeks (ng/mL)	9.8 (2.6)	9.5 (3.1)	10.9 (3.0)	0.324
	CsA at best eGFR (ng/mL)	262.3 (109.4)	273.5 (99.9)	284.4 (102.5)	0.386
	CsA at 1 week (ng/mL)	268.6 (102.3)	292.0 (95.8)	287.7 (95.5)	0.179
	CsA at 2 weeks (ng/mL)	252.6 (95.0)	227.2 (96.8)	292.5 (129.3)	0.175
	CsA at 3 weeks (ng/mL)	235.7 (86.7)	284.1 (123.8)	229.8 (88.7)	0.087
	TACER at best eGFR (ng/mL)	7.4 (2.7)	7.9 (3.5)	7.5 (2.1)	0.811
	TACER at 1 week (ng/mL)	7.8 (3.0)	8.3 (3.5)	10.3 (8.6)	0.812
	TACER at 2 weeks (ng/mL)	7.5 (2.3)	8.9 (3.4)	7.3 (0.8)	0.452
	TACER at 3 weeks (ng/mL)	7.5 (2.0)	8.5 (1.7)	7.4 (2.8)	0.232
Pathological findings at protocol biopsy at 1 month after kidney transplantation (%)	Recurrence of nephritis (%)	4 (4.3)	2 (3.1)	1 (2.6)	**0.013**
	Calcineurin inhibitor toxicity (%)	81 (8.7)	7 (10.9)	2 (5.3)	0.617
*De novo* DSA (%)		103 (11.0)	22 (36.7)	4 (11.8)	**< 0.001**
Rejection (pathological and clinical, %)		30 (2.8)	7 (9.6)	2 (4.8)	**0.007**
Graft survival period (months, SD)		74.4 (44.5)	72.3 (40.1)	66.5 (40.1)	0.592
Recipient death (%)		0	10 (13.7)	42 (100.0)	**<0.001**

Cr, creatine; CsA, cyclosporine A; DSA, donor-specific anti-human leukocyte antigen antibody; eGFR, estimated glomerular filtration rate; EVR, everolimus; HbA1c, hemoglobin A1c; HLA, human leukocyte antigen; ICFA, immunocomplex capture fluorescence analysis; IVIG, intravenous immunoglobulin; MMF, mycophenolate mofetil; MZ, mizoribine; PRA, panel reactive antibody; SD, standard deviation; TAC, tacrolimus; TACER, extended-release tacrolimus; Tc-99m DTPA, technetium-99 m diethylene triamine pentaacetic acid.

Preoperative flow cytometry B cell crossmatch after desensitization for preformed DSA became false positive when rituximab was administered. ICFA classes I and II were examined for recipients who received rituximab. The bold font indicates statistically significant results.

Regarding the recipient characteristics, significant differences were observed in recipient age (*p* < 0.001); cause of end-stage renal disease (*p* = 0.019); follow-up period (*p* < 0.001); transplantation from first-degree relative donors (*p* = 0.001); preoperative flow cytometry B cell crossmatch positivity (*p* = 0.010); dialysis vintage (*p* < 0.001); HLA-AB mismatch (*p* = 0.010); calcineurin inhibitor administration at KT (*p* < 0.001); calcineurin inhibitor administration at the best eGFR within 3 weeks after KT (*p* < 0.001); calcineurin inhibitor administration at 1, 2, and 3 weeks after KT (*p* < 0.001, *p* < 0.001, *p* < 0.001, respectively); mycophenolate mofetil, mizoribine, or everolimus administration at transplantation (*p* = 0.049); actual best eGFR within 3 weeks (*p* = 0.039); actual eGFRs at 1 and 2 weeks and 3, 6, and 12 months (*p* = 0.038, *p* = 0.013, *p* = 0.025, *p* < 0.001, and *p* < 0.001, respectively); recurrence of nephritis (*p* = 0.013); *de novo* DSA (*p* < 0.001); rejection (*p* = 0.007); and recipient death (*p* < 0.001).

#### 3.2.2. Operative outcomes of the donor and recipients

The operative outcomes of the donors and recipients are presented in Table 2. In the donor operation, significant differences were observed in donor nephrectomy operation time (*p* = 0.020) and operation methods (*p* < 0.001). In the recipient operation, significant differences were observed in cold ischemia time (*p* < 0.001), delayed graft function (*p* < 0.001), and occurrence of arterial thrombosis (*p* = 0.001), lymphocele (*p* < 0.001), incisional hernia (*p* = 0.007), and severe pneumonia (*p* = 0.001).

**Table 2:** Donor and recipient operative outcomes.

		Functioning graft	Graft loss	Death with functioning graft	*p-*value
		*n* = 1,059	*n* = 73	*n* = 42	
Donor operation					
Kidney laterality (left, %)		973 (91.9)	67 (91.8)	37 (88.1)	0.683
Kidney weight (g, SD)		117.2 (42.2)	183.5 (41.1)	182.1 (45.3)	0.217
Warm ischemia time (s, SD)		139.9 (69.4)	148.3 (72.0)	146.6 (45.8)	0.321
Operating time (min, SD)		208.4 (95.7)	218.1 (45.5)	214.1 (51.9)	**0.020**
Operation blood loss (mL, SD)		34.5 (12.7)	44.6 (13.5)	72.6 (18.9)	0.426
Adverse events	Arterial injury (%)	1 (0.1)	0	0	0.947
	Venous injury (%)	2 (0.2)	0	0	0.897
	Open conversion (%)	3 (0.3)	0	0	0.849
	Intraoperative bleeding (%)	2 (0.2)	0	0	0.897
	Subcapsular hematoma (%)	2 (0.2)	0	0	0.897
	Bowel injury (%)	1 (0.1)	0	0	0.947
Operation methods of donor nephrectomy	Hand-assisted laparoscopic (%)	1,011 (95.5)	64 (87.7)	34 (81.0)	**<0.001**
	Non-hand-assisted retroperitoneoscopic (%)	35 (3.3)	4 (5.5)	5 (11.9)	
	Open (%)	13 (1.2)	5 (6.8)	3 (7.1)	
Recipient operation					
Cold ischemia time (min, SD)		95.5 (39.0)	109.3 (47.8)	116.2 (43.5)	**<0.001**
Arterial reconstruction or ligation of thin upper pole artery (%)		300 (28.3)	20 (27.4)	16 (38.1)	0.378
Recipient perioperative adverse events	Delayed graft function (%)	0	0	1 (2.4)	**<0.001**
	Surgical site infection (%)	12 (1.1)	2 (2.7)	1 (2.4)	0.403
	Arterial thrombosis (%)	0	1 (1.4)	0	**0.001**
	Arterial stenosis (%)	2 (0.2)	0	0	0.897
	Urine leakage (%)	10 (0.9)	0	1 (2.4)	0.442
	Ureteral necrosis (%)	2 (0.2)	0	0	0.897
	Ureteral stenosis (%)	3 (0.3)	1 (1.4)	0	0.283
	Lymphocele (%)	9 (0.8)	5 (6.8)	0	**<0.001**
	Incisional hernia (%)	3 (0.3)	2 (2.7)	0	**0.007**
	Postoperative bleeding requiring reoperation (%)	14 (1.3)	1 (1.4)	2 (4.8)	0.187
	Gastrointestinal bleeding or perforation (%)	2 (0.2)	0	0	0.897
	Colon perforation (%)	3 (0.3)	0	0	0.849
	Severe pneumonia (%)	0	1 (1.4)	0	**0.001**

SD, standard deviation. The bold font isndicates statistically significant results.

#### 3.2.3. Causes of graft loss and death with functioning grafts

Graft loss was identified in 73 recipients (30, 18, 10, 8, 5, 1, and 1 cases of rejection, allograft nephropathy, infection, recurrent nephritis, cardiac events, arterial thrombosis, and unknown cause, respectively). Death with functioning grafts was observed in 42 recipients (13, 10, 4, 3, 3, and 9 cases of malignant diseases, cardiovascular diseases, accidents, infectious diseases, cerebrovascular diseases, and other causes, respectively).

#### 3.2.4. Impact of actual eGFR on graft loss

The results of the univariate Fine–Gray competing risk regression model for graft loss are presented in Supplementary Table S2. Significant differences were observed in male recipient (*p* = 0.032); preformed DSA (*p* = 0.013); preoperative desensitization (preoperative rituximab administration or splenectomy, preoperative double-filtration plasmapheresis, plasmapheresis, or intravenous immunoglobulin, *p* = 0.028); actual eGFR at 6 months after LDKT (*p* = 0.003); actual eGFR at 12 months after LDKT (*p* < 0.001); and donor age (*p* = 0.048). Table 3 and Supplementary Tables S3A–H show the graft loss risk of the actual best eGFR within 3 weeks after LDKT and actual eGFRs at 1, 2, and 3 weeks and at 1, 3, 6, and 12 months after LDKT adjusted for male recipient, preformed DSA, preoperative desensitization, and donor age using the multivariate Fine–Gray competing risk regression model. Significant differences were observed in the actual eGFRs at 6 and 12 months after LDKT (*p* = 0.015, hazard ratio [HR]: 0.946, 95% confidence interval [CI]: 0.904–0.989, *p* < 0.001; HR: 0.937, 95% CI: 0.907–0.967).

**Table 3:** Multivariate Fine–Gray competing model analysis for graft loss adjusted for male recipient, preformed DSA, preoperative desensitization, and donor age.

	*p*-value	Hazard ratio	95% confidence interval	
			Lower limit	Upper limit
Actual best eGFR within 3 weeks after transplantation (mL/min/1.73 m^2^)	0.930	1.001	0.980	1.022
Actual eGFR at 1 week after transplantation (mL/min/1.73 m^2^)	>0.999	1.000	0.978	1.022
Actual eGFR at 2 weeks after transplantation (mL/min/1.73 m^2^)	0.850	1.002	0.978	1.027
Actual eGFR at 3 weeks after transplantation (mL/min/1.73 m^2^)	0.810	0.996	0.967	1.027
Actual eGFR at 1 month after transplantation (mL/min/1.73 m^2^)	0.530	0.990	0.960	1.022
Actual eGFR at 3 months after transplantation (mL/min/1.73 m^2^)	0.470	0.986	0.947	1.025
Actual eGFR at 6 months after transplantation (mL/min/1.73 m^2^)	**0.015**	0.946	0.904	0.989
Actual eGFR at 12 months after transplantation (mL/min/1.73 m^2^)	**<0.001**	0.937	0.907	0.967

DSA, donor-specific antibody; eGFR, estimated glomerular filtration rate. The bold font indicates statistically significant results.

#### 3.2.5. Development of eGFR prediction models

Details of the recipients who met the inclusion and exclusion criteria for developing prediction models are presented in Supplementary Tables S4, S5. For developing prediction models, recipients with conversion of the immunosuppressive regimen, recurrence of nephritis, calcineurin inhibitor toxicity, rejection, operative adverse events in donor and recipient operations, and arterial reconstruction or ligation of the thin upper pole artery were excluded, and those with these factors were not identified as recipients for prediction models.

The donor and recipient characteristics and operative outcomes for the training and validation sets are presented in Supplementary Tables S6, S7. Significant differences were identified in donor sex (*p* = 0.042) and preoperative flow cytometry T cell crossmatch (*p* = 0.033). Supplementary Table S8 presents the training set results using 10-fold cross-validation for the ideal best eGFR within 3 weeks after LDKT. Model 4 had the best R and R-squared values (0.646 and 0.418, respectively). The best prediction model for the ideal best eGFR within 3 weeks after LDKT is presented in Supplementary Table S9. Additionally, the R and R-squared values in the validation set were 0.651 and 0.423, respectively (Table 4). Supplementary Table S10 presents the training set results using 10-fold cross-validation for the predicted ideal eGFR at 1 week after LDKT. Model 7 had the best R and R-squared values (0.573 and 0.328, respectively). Supplementary Table S11 shows the best prediction model for the predicted ideal eGFR at 1 week after LDKT, and the R and R-squared values in the validation set were 0.600 and 0.360, respectively (Table 4). Supplementary Table S12 shows the training set results using 10-fold cross-validation for the predicted ideal eGFR at 2 weeks after LDKT. Model 7 had the best R and R-squared values (0.619 and 0.383, respectively). Furthermore, the best-estimated model for the predicted ideal eGFR at 2 weeks after LDKT is presented in Supplementary Table S13. The R and R-squared values in the validation set were 0.598 and 0.358, respectively (Table 4). Supplementary Table S14 presents the training set results using 10-fold cross-validation for the predicted ideal eGFR at 3 weeks after LDKT, and model 7 had the best R and R-squared values (0.693 and 0.480, respectively). The best-estimated model for the predicted ideal eGFR at 3 weeks after LDKT is presented in Supplementary Table S15. Furthermore, the R and R-squared values in the validation set were 0.617 and 0.380, respectively (Table 4).

**Table 4:** Coefficients in the validation set.

	Model	R	R-squared
Best eGFR within 3 weeks after transplantation	4	0.651	0.423
eGFR at 1 week after transplantation	7	0.600	0.360
eGFR at 2 weeks after transplantation	7	0.598	0.358
eGFR at 3 weeks after transplantation	7	0.617	0.380

eGFR, estimated glomerular filtration rate. The bold font indicates statistically significant results.

#### 3.2.6. Impact of predicted ideal and actual eGFRs on graft loss

Supplementary Figures S1A–D shows the association between the perioperative predicted ideal and actual eGFRs.

The results of the univariate Fine–Gray competing risk regression model for graft loss are presented in Supplementary Table S16. Significant differences were observed in male recipient (*p* = 0.032); preformed DSA (*p* = 0.013); preoperative desensitization (*p* = 0.028); predicted ideal best eGFR/actual best eGFR within 3 weeks after LDKT (*p* < 0.001); predicted ideal eGFRs/actual eGFRs at 1, 2, and 3 weeks after LDKT (*p* = 0.045, *p* = 0.008, and *p* < 0.001, respectively); and donor age (*p* = 0.048). Table 5 and Supplementary Tables S17A–D show the graft loss risk of the predicted ideal best eGFR/actual best eGFR within 3 weeks after LDKT and predicted ideal eGFRs/actual eGFRs at 1, 2, and 3 weeks after LDKT adjusted for male recipient, preformed DSA, preoperative desensitization, and donor age using the multivariate Fine–Gray competing risk regression model. Additionally, significant differences were identified in the predicted ideal best eGFR/actual best eGFR within 3 weeks after LDKT and the predicted ideal eGFRs/actual eGFRs at 1, 2, and 3 weeks after LDKT (*p* < 0.001, HR: 1.496, 95% CI: 1.225–1.826; *p* = 0.006, HR: 1.309, 95% CI: 1.079–1.588; *p* = 0.002, HR: 1.323, 95% CI: 1.105–1.584; and *p* < 0.001, HR: 1.452, 95% CI: 1.240–1.699, respectively). In Supplementary Tables S17A–D, in addition to the significant differences in predicted ideal eGFRs/actual eGFRs, significant differences were observed in male recipient, preformed DSA, and donor age.

**Table 5:** Multivariate Fine–Gray competing model analysis for graft loss adjusted for male recipient, preformed DSA, preoperative desensitization, and donor age.

	*p*-value	Hazard ratio	95% confidence interval	
			Lower limit	Upper limit
Predicted ideal best eGFR/actual best eGFR within 3 weeks after transplantation	**<0.001**	1.496	1.225	1.826
Predicted ideal eGFR/actual eGFR at 1 week after transplantation	**0.006**	1.309	1.079	1.588
Predicted ideal eGFR/actual eGFR at 2 weeks after transplantation	**0.002**	1.323	1.105	1.584
Predicted ideal eGFR/actual eGFR at 3 weeks after transplantation	**<0.001**	1.452	1.240	1.699

DSA, donor-specific anti-human leukocyte antigen antibody; eGFR, estimated glomerular filtration rate. The bold font indicates statistically significant results.

## 4. Discussion

This study suggests that the actual eGFRs at 6 and 12 months after LDKT could be an independent risk factor for graft loss, although the actual eGFR within 3 months after LDKT does not seem to be a risk factor. However, the predicted ideal best eGFR/actual best eGFR within 3 weeks after LDKT and the predicted ideal eGFRs/actual eGFRs at 1, 2, and 3 weeks after LDKT might be independent prognostic factors for graft loss.

In this work, as LDKTs were performed between Asian (Japanese) recipients and donors, the race composition differed from those reported previously, and the BMI values of the recipients and donors were lower than those reported in previous works from different countries (24–26). In Japan, LDKT is limited between relatives. This may have contributed to the higher rate of ABO-incompatible KTs (33.4%) found in this study than those reported in previous studies on LDKT, although the rate of preformed-DSA KTs (7.1%) was similar to those reported in previous studies on LDKT (27, 28). The desensitization protocols for ABO-incompatible and preformed-DSA KTs were similar to those of previous reports, although those for preformed-DSA KT have not been established (28–30). However, the *de novo* DSA, rejection, graft loss, and death with functioning graft rates during the median observation period of 77.0 months were 11.0, 3.3, 6.2, and 3.6%, respectively. Interestingly, these results are similar to those of previous reports from other countries (31–33). In this study, the routine hospital stay at our institution after the transplantation was 3 weeks, which might be longer than that in other countries (34, 35). This might have facilitated a more in-depth investigation of post-LDKT graft function.

The eGFR at 1 year after KT could be a prognostic factor for graft loss (2, 10–17). However, no studies to date have investigated the impact of eGFR within 1 year of KT. This study is the first to examine the effects of actual eGFRs within 1 year on graft loss. Using multivariate Fine–Gray competing model analysis, actual eGFRs at 6 and 12 months after LDKT, preformed DSA, and male recipient were shown to be independent prognostic factors for graft loss. In previous studies, the graft survival of recipients with preformed DSAs was worse than that of those without because of antibody-mediated rejection (AMR). However, desensitization was performed to prevent acute AMR (28, 29, 31). Although many clinical studies on desensitization using intravenous immunoglobulin, rituximab, plasmapheresis, and imlifidase have been conducted to improve the graft survival of recipients with preformed DSAs, no desensitization regimen for preformed DSAs has been established (36–39). Here, the recipients with preformed DSAs were desensitized using rituximab, plasmapheresis, and intravenous immunoglobulin administration. However, these desensitization procedures were ineffective in improving graft survival. Consistent with previous studies, male recipient was also found to be an independent prognostic factor for graft loss (40, 41). This study is novel because it investigated the impact of the actual eGFRs on graft loss within 1 year after LDKT. Moreover, no studies have investigated the impact of the actual eGFRs at 1, 2, and 3 weeks and 1, 3, and 6 months after LDKT on graft survival (2, 10–15). Therefore, this study revealed that actual eGFRs within 3 months after LDKT could not be an independent prognostic factor for graft loss.

Notably, the prediction models for ideal eGFRs at 1, 2, and 3 weeks after LDKT and the ideal best eGFR within 3 weeks were developed using 10-fold cross-validation and stepwise multiple regression model analysis. Ideal KTs were selected by excluding problematic donors and recipients during the perioperative period. Finally, data from 676 recipients were used to develop prediction models for ideal eGFRs during this period. This study is novel because no studies to date have investigated prediction models for ideal eGFRs during the perioperative period. During the development of the prediction models, the trough levels of tacrolimus and extended-release tacrolimus were separately presented, as they were found to be significantly different when the same dose was administered in a previous study (42).

Overfitting of the model was prevented using 10-fold cross-validation (43, 44). The predicted ideal best eGFR/actual best eGFR within 3 weeks after LDKT and predicted ideal eGFRs/actual eGFRs at 1, 2, and 3 weeks after LDKT were obtained in 1174 recipients to investigate the impact of ideal eGFRs/actual eGFRs on graft loss. In the multivariate Fine–Gray competing model analysis, covariates that were independent prognostic factors in the univariate Fine–Gray competing model analysis were used as follows: male recipient; preformed DSA; preoperative desensitization; donor age; predicted best eGFR/actual best eGFR within 3 weeks after LDKT; and predicted ideal eGFRs/actual eGFRs at 1, 2, and 3 weeks after LDKT. In the multivariate Fine–Gray competing model analysis, in addition to the predicted ideal best eGFR/actual best eGFR within 3 weeks after LDKT and predicted ideal eGFRs/actual eGFRs at 1, 2, and 3 weeks after LDKT, male recipient, preformed DSA, and donor age were independent prognostic factors for graft loss (3, 28, 29, 31, 40, 41). In this analysis, male recipient and preformed DSA were the prognostic factors for graft loss, similar to those indicated by the multivariate analyzes for the impact of actual eGFRs on graft loss. Additionally, donor age was found to be an independent prognostic factor for graft loss. This result is consistent with those of previous studies indicating that graft loss may occur more frequently when the graft is transplanted from elderly donors owing to donor age-related graft nephrosclerosis (3, 41, 45). Furthermore, the graft loss risk of the predicted ideal eGFRs/actual eGFRs adjusted with the multivariate Fine–Gray competing model analysis using male recipient, preformed DSA, and donor age as factors was significant. These results show that the graft loss risk increases as the predicted ideal eGFR/actual eGFR increases. Moreover, this implies that when recipients receive actual eGFRs that are lower than those of the predicted ideal eGFR, the graft survival may be worse than that of recipients who obtained better eGFRs than the predicted ideal eGFR. Although actual eGFRs at 1, 2, and 3 weeks after LDKT were not significant predictors for graft loss, those at 6 and 12 months after LDKT were significant predictors. This implies that we cannot predict graft loss based on the actual eGFRs at 1, 2, and 3 weeks after KT. The *P*-values in the multivariate Fine–Gray competing model analysis for graft loss, adjusted for male recipient, preformed DSA, preoperative desensitization, and donor age, decreased as the time after KT passed. This may imply that the widening disparities of the actual eGFR after KT between the graft loss and non-graft loss groups did not contribute to graft loss prediction until 6 months after KT. However, it may be useful to reveal the predictor for graft loss earlier and implement measures based on the results. Therefore, the predicted ideal eGFR/actual eGFR was successfully developed to make the eGFRs at 1, 2, and 3 weeks more useful predictors for graft loss. This finding enabled the detection of slightly widening disparities of eGFR after KT between the graft loss and non-graft loss groups, which could not be detected using the actual eGFR after KT.

Many factors might prevent recipients from obtaining an ideal eGFR during the perioperative period, including rejection, delayed graft function, and operative complications (7, 46, 47). However, the period of 3 weeks after LDKT is early to optimize immunosuppression, treat comorbidities, and detect and treat surgical complications, including graft vascular stenosis and urinary tract obstruction. Therefore, to obtain the ideal eGFR, preventing rejection, delayed graft function, and intraoperative surgical complications, which may negatively affect graft function within 3 weeks after LDKT, might be crucial. These results are novel because long-term graft survival can be predicted using prediction models for perioperative ideal eGFRs. Accordingly, this study demonstrated the importance of obtaining an ideal eGFR during the perioperative period.

Furthermore, considering the recent advancements in artificial intelligence technology, the development of tools for predicting eGFR after KT using data from large-scale multicenter studies is expected. Therefore, this study’s results, which suggest the potential utility of a predictive tool for the ideal eGFR rather than relying solely on the actual eGFR, can lead to the advancement of innovative studies in the field of KT.

The retrospective design of the study and the fact that it was conducted in a single institution to examine the impact of predicted eGFRs on graft survival were limitations of this work. Therefore, a prospective multicenter randomized study focusing on the effects of predicted ideal eGFRs/actual eGFRs on graft survival should be conducted to verify the results and elucidate the causes of failure to obtain ideal eGFRs.

In conclusion, the predicted ideal eGFRs/actual eGFRs at 1, 2, and 3 weeks after LDKT and the predicted ideal best eGFR/actual best eGFR within 3 weeks may forecast graft survival after adult LDKT. Therefore, obtaining an ideal perioperative eGFR is crucial for improving long-term graft survival.

## Data availability statement

The raw data supporting the conclusions of this article will be made available by the authors, without undue reservation.

## Ethics statement

The studies involving humans were approved by Nagoya Daini Red Cross Hospital’s Institutional Review Board (Aichi, Japan; approval number: 1504). The studies were conducted in accordance with the local legislation and institutional requirements. Written informed consent for participation was not required from the participants or the participants’ legal guardians/next of kin in accordance with the national legislation and institutional requirements.

## Author contributions

TH designed and acquired the data, interpreted the results, and drafted the manuscript. YH, MO, YM, AT, and TK acquired the data. KF, NG, TI, and SN interpreted the results. KU and YW approved the final version of the manuscript. All authors contributed to the article and approved the submitted version.

## Conflict of interest

The authors declare that the research was conducted in the absence of any commercial or financial relationships that could be construed as a potential conflict of interest.

## Publisher’s note

All claims expressed in this article are solely those of the authors and do not necessarily represent those of their affiliated organizations, or those of the publisher, the editors and the reviewers. Any product that may be evaluated in this article, or claim that may be made by its manufacturer, is not guaranteed or endorsed by the publisher.

## Supplementary material

The Supplementary material for this article can be found online at: https://www.frontiersin.org/articles/10.3389/fmed.2023.1187777/full#supplementary-material
