# Supplementary material for: Prediction models for the recipients’ ideal perioperative estimated glomerular filtration rates for predicting graft survival after adult living-donor kidney transplantation
Source: Front Med (Lausanne). 2023 Aug 31;10:1187777. doi: 10.3389/fmed.2023.1187777 (PMC10501755; doi:10.3389/fmed.2023.1187777)
Supplement: Supplementary file 1 [file Data_Sheet_1.docx]

Supplementary Material

Prediction Models for the Recipients’ Ideal Perioperative Estimated Glomerular Filtration Rates for Predicting Graft Survival after Adult Living-donor Kidney Transplantation

Takahisa Hiramitsu^1*^, Yuki Hasegawa^1^, Kenta Futamura^1^, Manabu Okada^1^, Yutaka Matsuoka^2^, Norihiko Goto^1^, Toshihiro Ichimori^1^, Shunji Narumi^1^, Asami Takeda^3^, Takaaki Kobayashi^4^, Kazuharu Uchida^2^, Yoshihiko Watarai^1^

^1^Department of Transplant and Endocrine Surgery, Japanese Red Cross Aichi Medical Center Nagoya Daini Hospital, 2-9 Myoken-cho, Showa-ku, Nagoya, Aichi, Japan

^2^Department of Renal Transplant Surgery, Masuko Memorial Hospital, 35-28 Takehashi-cho, Nakamura-ku, Nagoya, Aichi, Japan

^3^Department of Nephrology, Japanese Red Cross Aichi Medical Center Nagoya Daini Hospital, 2-9 Myoken-cho, Showa-ku, Nagoya, Aichi, Japan

^4^Department of Renal Transplant Surgery, Aichi Medical University School of Medicine, 1-1 Yazakokarimata, Nagakute, Aichi, Japan

*** Correspondence:** Takahisa Hiramitsu; [thira@nagoya2.jrc.or.jp](mailto:thira@nagoya2.jrc.or.jp)

## Supplementary Figures


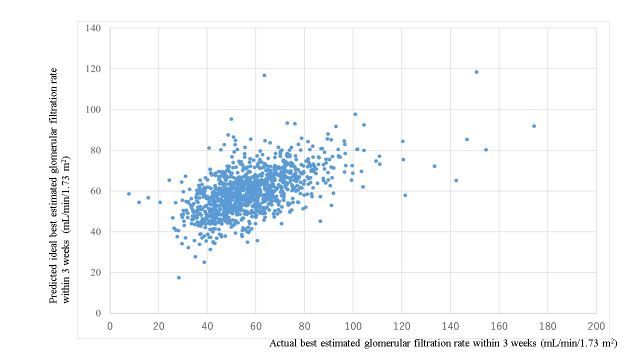


**Supplementary Figure S1 (A).** Association between the perioperative predicted ideal best eGFR and actual best eGFR within 3 weeks after kidney transplantation. eGFR, estimated glomerular filtration rate

**
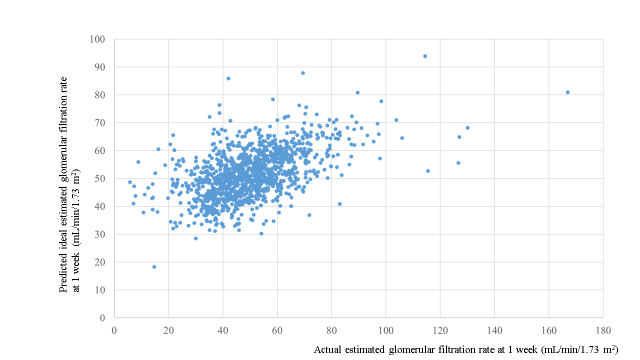
**

**Supplementary Figure S1 (B).** Association between the perioperative predicted ideal eGFR and actual eGFR at 1 week after kidney transplantation. eGFR, estimated glomerular filtration rate

**
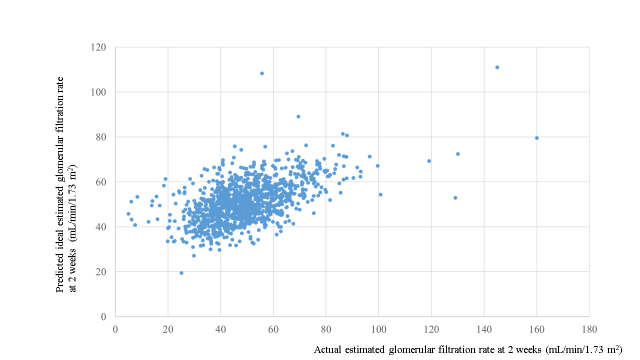
**

**Supplementary Figure S1 (C).** Association between the perioperative predicted ideal eGFR and actual eGFR at 2 weeks after kidney transplantation. eGFR, estimated glomerular filtration rate

**
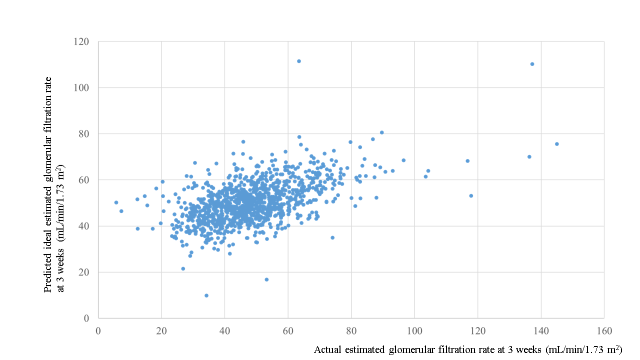
**

**Supplementary Figure S1 (D).** Association between the perioperative predicted ideal and actual eGFRs at 3 weeks after kidney transplantation. eGFR, estimated glomerular filtration rate

## Supplementary Tables

| **Supplementary Table S1.** Detailed reasons for excluding recipients from the development of prediction models | | | | | |
| --- | --- | --- | --- | --- | --- |
|  |  | Functioning graft | Graft loss | Death with functioning graft | *P-*value |
|  |  | n=438 | n=39 | n=21 |  |
| **DONOR** |  |  |  |  |  |
| Intraoperative adverse events | Arterial injury(%) | 1 (0.2) | 0 | 0 | 0.934 |
|  | Venous injury (%) | 2 (0.5) | 0 | 0 | 0.872 |
|  | Open conversion (%) | 3 (0.7) | 0 | 0 | 0.813 |
|  | Intraoperative bleeding (%) | 2 (0.5) | 0 | 0 | 0.872 |
|  | Subcapsular hematoma (%) | 2 (0.5) | 0 | 0 | 0.872 |
|  | Bowel injury (%) | 1 (0.2) | 0 | 0 | 0.934 |
| **RECIPIENT** |  |  |  |  |  |
| Arterial reconstruction or ligation of thin upper pole artery (%) |  | 300 (68.5) | 20 (51.3) | 16 (76.2) | 0.061 |
| Recipient perioperative adverse events | Delayed graft function (%) | 0 | 0 | 1 (4.8) | **<0.001** |
|  | Surgical site infection (%) | 12 (2.7) | 2 (5.1) | 1 (4.8) | 0.628 |
|  | Arterial thrombosis (%) | 0 | 1 (2.6) | 0 | **0.003** |
|  | Arterial stenosis (%) | 2 (0.5) | 0 | 0 | 0.872 |
|  | Urine leakage (%) | 10 (2.3) | 0 | 1 (4.8) | 0.466 |
|  | Ureteral necrosis (%) | 2 (0.5) | 0 | 0 | 0.872 |
|  | Ureteral stenosis (%) | 3 (0.7) | 1 (2.6) | 0 | 0.414 |
|  | Lymphocele (%) | 9 (2.1) | 5 (12.8) | 0 | **<0.001** |
|  | Incisional hernia (%) | 3 (0.7) | 2 (5.1) | 0 | **0.026** |
|  | Postoperative bleeding requiring reoperation (%) | 14 (3.2) | 1 (2.6) | 2 (9.5) | 0.283 |
|  | Gastrointestinal bleeding or perforation (%) | 2 (0.5) | 0 | 0 | 0.872 |
|  | Colon perforation (%) | 3 (0.7) | 0 | 0 | 0.813 |
|  | Sever pneumonia (%) | 0 | 1 (2.6) | 0 | **0.003** |
| Conversion of immunosuppressive regimen within 1 month (%) |  | 13 (3.0) | 2 (5.1) | 0 | 0.534 |
| Pathological findings at protocol biopsy at 1 month after kidney transplantation (%) | Recurrence of nephritis (%) | 4 (0.9) | 2 (5.1) | 1 (4.8) | **0.041** |
|  | Calcineurin inhibitor toxicity (%) | 81 (18.5) | 7 (17.9) | 2 (9.5) | 0.522 |
| Rejection (pathological and clinical, %) |  | 30 (6.8) | 7 (17.9) | 2 (9.5) | 0.055 |
| The bold font indicates statistically significant results. | | | | | |

| **Supplementary Table S2.** Univariate Fine–Grey competing model analysis for graft loss | | | | | |
| --- | --- | --- | --- | --- | --- |
|  |  | *P-*value | Hazard ratio | 95% confidence interval | |
|  |  |  |  | Lower limit | Upper limit |
| **RECIPIENT CHARACTERISTICS** |  |  |  |  |  |
| Recipient age (years) |  | 0.470 | 0.993 | 0.976 | 1.011 |
| Male recipient (vs. female) |  | **0.032** | 1.754 | 1.050 | 2.932 |
| Recipient body mass index (kg/m^2^) |  | 0.150 | 1.042 | 0.985 | 1.103 |
| Transplantation from a first-degree relative donor (vs. non-transplantation from a first-degree relative donor) |  | 0.080 | 1.514 | 0.952 | 2.406 |
| Preoperative flow cytometry T cell crossmatch positive (vs. negative) |  | 0.770 | 1.232 | 0.299 | 5.070 |
| Preoperative flow cytometry B cell crossmatch positive (vs. negative) |  | 0.063 | 1.749 | 0.971 | 3.152 |
| Dialysis vintage (months) |  | 0.530 | 0.999 | 0.997 | 1.002 |
| Preoperative sensitization (transfusion, pregnancy, transplantation) (vs. non-preoperative sensitization) |  | 0.140 | 0.698 | 0.431 | 1.130 |
| HLA-AB mismatch |  | 0.440 | 0.917 | 0.736 | 1.143 |
| HLA-DR mismatch |  | 0.520 | 0.889 | 0.620 | 1.274 |
| Preoperative PRA class I positive (≥5%) (vs. negative) |  | 0.330 | 1.356 | 0.733 | 2.510 |
| Preoperative PRA class II positive (≥5%) (vs. negative) |  | 0.860 | 0.914 | 0.333 | 2.506 |
| ABO incompatible transplantation (vs. non-ABO incompatible transplantation) |  | 0.300 | 1.284 | 0.801 | 2.060 |
| Preformed DSA (vs. non-preformed DSA) |  | **0.013** | 2.260 | 1.185 | 4.311 |
| Preoperative desensitization (preoperative rituximab administration or splenectomy, preoperative double filtration plasmapheresis, plasmapheresis, or IVIG) (vs. non-preoperative desensitization) |  | **0.028** | 1.670 | 1.056 | 2.639 |
| Calcineurin inhibitor administration at transplantation |  | 0.304 | (for all categories) |  |  |
|  | TAC | ref | 1.000 |  |  |
|  | CsA | 0.790 | 0.933 | 0.556 | 1.565 |
|  | TACER | 0.140 | 0.534 | 0.232 | 1.229 |
| MMF, MZ, or EVR administration at transplantation |  | 0.083 | (for all categories) |  |  |
|  | MMF | ref | 1.000 |  |  |
|  | MZ | 0.170 | 0.396 | 0.105 | 1.494 |
|  | EVR | 0.070 | 0.341 | 0.106 | 1.090 |
| Preoperative ejection fraction on ultrasonographic cardiography (%) |  | 0.690 | 0.993 | 0.962 | 1.026 |
| Preoperative ventricular wall motion asynergy on ultrasonographic cardiography (vs. non-preoperative ventricular wall motion asynergy on ultrasonographic cardiography) |  | 0.230 | 1.479 | 0.777 | 2.815 |
| Actual best eGFR within 3 weeks (mL/min/1.73 m^2^) |  | 0.440 | 0.991 | 0.970 | 1.014 |
| Actual eGFR at 1 week (mL/min/1.73 m^2^) |  | 0.500 | 0.992 | 0.971 | 1.015 |
| Actual eGFR at 2 weeks (mL/min/1.73 m^2^) |  | 0.500 | 0.992 | 0.968 | 1.016 |
| Actual eGFR at 3 weeks (mL/min/1.73 m^2^) |  | 0.280 | 0.984 | 0.956 | 1.013 |
| Actual eGFR at 1 month (mL/min/1.73 m^2^) |  | 0.120 | 0.978 | 0.951 | 1.006 |
| Actual eGFR at 3 months (mL/min/1.73 m^2^) |  | 0.110 | 0.973 | 0.942 | 1.006 |
| Actual eGFR at 6 months (mL/min/1.73 m^2^) |  | **0.003** | 0.942 | 0.907 | 0.980 |
| Actual eGFR at 12 months (mL/min/1.73 m^2^) |  | **<0.001** | 0.933 | 0.905 | 0.962 |
| **DONOR CHARACTERISTICS** |  |  |  |  |  |
| Donor age (years) |  | **0.048** | 1.027 | 1.000 | 1.055 |
| Male donor (vs. female) |  | >0.999 | 1.001 | 0.623 | 1.608 |
| Donor body mass index (kg/m^2^) |  | 0.370 | 1.038 | 0.957 | 1.126 |
| Baseline biopsy findings at 1 h after transplantation (vs. non-baseline biopsy findings at 1 h after transplantation) |  | 0.910 | 1.027 | 0.645 | 1.634 |
| Preoperative comorbidities ≥1 (vs. non-preoperative comorbidity) |  | 0.090 | 1.683 | 0.922 | 3.073 |
| Smoking history (vs. non-smoking history) |  | 0.490 | 1.108 | 0.830 | 1.479 |
| Preoperative eGFR (mL/min/1.73 m^2^) |  | 0.620 | 0.996 | 0.980 | 1.012 |
| **OPERATION FACTORS** |  |  |  |  |  |
| Kidney laterality (left) (vs. right) |  | 0.900 | 1.056 | 0.447 | 2.492 |
| Kidney weight (g) |  | 0.060 | 1.004 | 1.000 | 1.009 |
| Warm ischemia time (s) |  | 0.810 | 1.000 | 0.997 | 1.003 |
| Donor operation time (min) |  | 0.280 | 0.998 | 0.994 | 1.002 |
| Donor operation blood loss (mL) |  | 0.610 | 1.000 | 0.999 | 1.002 |
| Arterial reconstruction or ligation of thin upper pole artery (vs. non-arterial reconstruction or ligation of thin upper pole artery) | | 0.920 | 0.973 | 0.582 | 1.626 |
| Split kidney function on Tc-99m DTPA scintigraphy (%) | | 0.150 | 1.043 | 0.985 | 1.105 |
| Operation methods of donor nephrectomy |  | 0.093 | (for all categories) |  |  |
|  | Hand-assisted laparoscopic | ref | 1.000 |  |  |
|  | Non-hand assisted retro-peritoneoscopic | 0.200 | 0.510 | 0.184 | 1.416 |
|  | Open | 0.110 | 2.194 | 0.848 | 5.676 |
| Cold ischemia time (min) |  | 0.450 | 1.002 | 0.997 | 1.006 |
| Abbreviations: CsA, cyclosporin A; DSA, donor-specific anti-human leukocyte antigen antibody; eGFR, estimated glomerular filtration rate; EVR, everolimus; HLA, human leukocyte antigen; IVIG, intravenous immunoglobulin; MMF, mycophenolate mofetil; MZ, mizoribine; PRA, panel reactive antibody; ref, reference; TAC, tacrolimus; TACER, extended-release tacrolimus; Tc-99m DTPA, technetium-99m diethylene triamine pentaacetic acid. The bold font indicates statistically significant results. | | | | | |

| **Supplementary Table S3 (A).** Multivariate Fine–Gray competing model analysis for graft loss using actual best eGFR within 3 weeks after transplantation | | | | |
| --- | --- | --- | --- | --- |
|  | *P*-value | Hazard ratio | 95% confidence interval | |
|  |  |  | Lower limit | Upper limit |
| Actual best eGFR within 3 weeks after transplantation (mL/min/1.73 m^2^) | 0.930 | 1.001 | 0.980 | 1.022 |
| Male recipient (vs. female) | **0.003** | 2.382 | 1.339 | 4.236 |
| Preformed DSA (vs. non-preformed DSA) | **0.006** | 2.780 | 1.348 | 5.734 |
| Preoperative desensitization (preoperative rituximab administration or splenectomy, preoperative double filtration plasmapheresis, plasmapheresis, or IVIG) (vs. non-preoperative desensitization) | 0.078 | 1.525 | 0.954 | 2.441 |
| Donor age (years) | 0.050 | 1.029 | 1.000 | 1.059 |
| Abbreviations: DSA, donor-specific anti-human leukocyte antigen antibody; eGFR, estimated glomerular filtration rate; IVIG, intravenous immunoglobulin. The bold font indicates statistically significant results. | | | | |

**Supplementary Table S3 (B).** Multivariate Fine–Gray competing model analysis for graft loss using actual eGFR at 1 week after transplantation

|  | | | | |
| --- | --- | --- | --- | --- |
|  | *P*-value | Hazard ratio | 95% confidence interval | |
|  |  |  | Lower limit | Upper limit |
| Actual eGFR at 1 week after transplantation (mL/min/1.73 m^2^) | >0.999 | 1.000 | 0.978 | 1.022 |
| Male recipient (vs. female) | **0.003** | 2.368 | 1.336 | 4.199 |
| Preformed DSA (vs. non-preformed DSA) | **0.006** | 2.773 | 1.342 | 5.733 |
| Preoperative desensitization (preoperative rituximab administration or splenectomy, preoperative double filtration plasmapheresis, plasmapheresis, or IVIG) (vs. non-preoperative desensitization) | 0.078 | 1.525 | 0.953 | 2.439 |
| Donor age (years) | **0.048** | 1.029 | 1.000 | 1.058 |
| Abbreviations: DSA, donor-specific anti-human leukocyte antigen antibody; eGFR, estimated glomerular filtration rate; IVIG, intravenous immunoglobulin. The bold font indicates statistically significant results. | | | | |

| **Supplementary Table S3 (C).** Multivariate Fine–Gray competing model analysis for graft loss using actual eGFR at 2 weeks after transplantation | | | | |
| --- | --- | --- | --- | --- |
|  | *P*-value | Hazard ratio | 95% confidence interval | |
|  |  |  | Lower limit | Upper limit |
| Actual eGFR at 2 weeks after transplantation (mL/min/1.73 m^2^) | 0.850 | 1.002 | 0.978 | 1.027 |
| Male recipient (vs. female) | **0.003** | 2.397 | 1.354 | 4.244 |
| Preformed DSA (vs. non-preformed DSA) | **0.006** | 2.778 | 1.343 | 5.748 |
| Preoperative desensitization (preoperative rituximab administration or splenectomy, preoperative double filtration plasmapheresis, plasmapheresis, or IVIG) (vs. non-preoperative desensitization) | 0.082 | 1.524 | 0.949 | 2.450 |
| Donor age (years) | **0.048** | 1.030 | 1.000 | 1.060 |
| Abbreviations: DSA, donor-specific anti-human leukocyte antigen antibody; eGFR, estimated glomerular filtration; IVIG, intravenous immunoglobulin. The bold font indicates statistically significant results. | | | | |

| **Supplementary Table S3 (D).** Multivariate Fine–Gray competing model analysis for graft loss using actual eGFR at 3 weeks after transplantation | | | | |
| --- | --- | --- | --- | --- |
|  | *P*-value | Hazard ratio | 95% confidence interval | |
|  |  |  | Lower limit | Upper limit |
| Actual eGFR at 3 weeks after transplantation (mL/min/1.73 m^2^) | 0.810 | 0.996 | 0.967 | 1.027 |
| Male recipient (vs. female) | **0.009** | 2.316 | 1.231 | 4.356 |
| Preformed DSA (vs. non-preformed DSA) | **0.004** | 3.037 | 1.430 | 6.450 |
| Preoperative desensitization (preoperative rituximab administration or splenectomy, preoperative double filtration plasmapheresis, plasmapheresis, or IVIG) (vs. non-preoperative desensitization) | 0.120 | 1.483 | 0.899 | 2.447 |
| Donor age (years) | 0.050 | 1.032 | 1.000 | 1.065 |
| Abbreviations: DSA, donor-specific anti-human leukocyte antigen antibody; eGFR, estimated glomerular filtration rate; IVIG, intravenous immunoglobulin. The bold font indicates statistically significant results. | | | | |

| **Supplementary Table S3 (E).** Multivariate Fine–Gray competing model analysis for graft loss using actual eGFR at 1 month after transplantation | | | | |
| --- | --- | --- | --- | --- |
|  | *P*-value | Hazard ratio | 95% confidence interval | |
|  |  |  | Lower limit | Upper limit |
| Actual eGFR at 1 month after transplantation (mL/min/1.73 m^2^) | 0.530 | 0.990 | 0.960 | 1.022 |
| Male recipient (vs. female) | **0.009** | 2.226 | 1.222 | 4.057 |
| Preformed DSA (vs. non-preformed DSA) | **0.008** | 2.706 | 1.304 | 5.612 |
| Preoperative desensitization (preoperative rituximab administration or splenectomy, preoperative double filtration plasmapheresis, plasmapheresis, or IVIG) (vs. non-preoperative desensitization) | 0.090 | 1.502 | 0.939 | 2.403 |
| Donor age (years) | 0.140 | 1.023 | 0.993 | 1.054 |
| Abbreviations: DSA, donor-specific anti-human leukocyte antigen antibody; eGFR, estimated glomerular filtration rate; IVIG, intravenous immunoglobulin. The bold font indicates statistically significant results. | | | | |

| **Supplementary Table S3 (F).** Multivariate Fine–Gray competing model analysis for graft loss using actual eGFR at 3 months after transplantation | | | | |
| --- | --- | --- | --- | --- |
|  | *P*-value | Hazard ratio | 95% confidence interval | |
|  |  |  | Lower limit | Upper limit |
| Actual eGFR at 3 months after transplantation (mL/min/1.73 m^2^) | 0.470 | 0.986 | 0.947 | 1.025 |
| Male recipient (vs. female) | **0.019** | 2.117 | 1.131 | 3.965 |
| Preformed DSA (vs. non-preformed DSA) | **0.009** | 2.707 | 1.284 | 5.710 |
| Preoperative desensitization (preoperative rituximab administration or splenectomy, preoperative double filtration plasmapheresis, plasmapheresis, or IVIG) (vs. non-preoperative desensitization) | 0.120 | 1.458 | 0.905 | 2.349 |
| Donor age (years) | 0.310 | 1.018 | 0.984 | 1.053 |
| Abbreviations: DSA, donor-specific anti-human leukocyte antigen antibody; eGFR, estimated glomerular filtration rate; IVIG, intravenous immunoglobulin. The bold font indicates statistically significant results. | | | | |

| **Supplementary Table S3 (G).** Multivariate Fine–Gray competing model analysis for graft loss using actual eGFR at 6 months after transplantation | | | | |
| --- | --- | --- | --- | --- |
|  | *P*-value | Hazard ratio | 95% confidence interval | |
|  |  |  | Lower limit | Upper limit |
| Actual eGFR at 6 months after transplantation (mL/min/1.73 m^2^) | **0.015** | 0.946 | 0.904 | 0.989 |
| Male recipient (vs. female) | 0.054 | 1.872 | 0.990 | 3.542 |
| Preformed DSA (vs. non-preformed DSA) | **0.018** | 2.552 | 1.177 | 5.533 |
| Preoperative desensitization (preoperative rituximab administration or splenectomy, preoperative double filtration plasmapheresis, plasmapheresis, or IVIG) (vs. non-preoperative desensitization) | 0.160 | 1.405 | 0.872 | 2.262 |
| Donor age (years) | >0.999 | 1.000 | 0.966 | 1.035 |
| Abbreviations: DSA, donor-specific anti-human leukocyte antigen antibody; eGFR, estimated glomerular filtration rate; IVIG, intravenous immunoglobulin. The bold font indicates statistically significant results. | | | | |

| **Supplementary Table S3 (H).** Multivariate Fine–Gray competing model analysis for graft loss using actual eGFR at 12 months after transplantation | | | | |
| --- | --- | --- | --- | --- |
|  | *P*-value | Hazard ratio | 95% confidence interval | |
|  |  |  | Lower limit | Upper limit |
| Actual eGFR at 12 months after transplantation (mL/min/1.73 m^2^) | **<0.001** | 0.937 | 0.907 | 0.967 |
| Male recipient (vs. female) | **0.033** | 2.007 | 1.060 | 3.800 |
| Preformed DSA (vs. non-preformed DSA) | **0.021** | 2.564 | 1.152 | 5.706 |
| Preoperative desensitization (preoperative rituximab administration or splenectomy, preoperative double filtration plasmapheresis, plasmapheresis, or IVIG) (vs. non-preoperative desensitization) | 0.250 | 1.324 | 0.820 | 2.138 |
| Donor age (years) | 0.870 | 0.997 | 0.967 | 1.029 |
| Abbreviations: DSA, donor-specific anti-human leukocyte antigen antibody; eGFR, estimated glomerular filtration rate; IVIG, intravenous immunoglobulin. The bold font indicates statistically significant results. | | | | |

| **Supplementary Table S4.** Donor and recipient characteristics | | | | | | | |
| --- | --- | --- | --- | --- | --- | --- | --- |
|  |  | Recipients for prediction models | Excluded recipients from prediction models | *P-*value | Odds ratio | 95% confidence interval | |
|  |  | n=676 | n=498 |  |  | Lower limit | Upper limit |
| **DONOR** |  |  |  |  |  |  |  |
| Donor age (years, SD) |  | 58.5 (10.0) | 59.5 (9.8) | **0.038** |  |  |  |
| Donor sex (male, %) |  | 223 (33.0) | 208 (41.8) | **0.002** | 1.457 | 1.147 | 1.852 |
| Donation to first-degree relative recipients (%) |  | 311 (46.0) | 229 (46.0) | 0.994 | 0.999 | 0.792 | 1.260 |
| Smoking history (%) |  | 277 (41.0) | 245 (49.2) | **0.005** | 1.395 | 1.105 | 1.761 |
| Preoperative comorbidities ≥1 (%) |  | 476 (70.4) | 385 (77.3) | **0.008** | 1.432 | 1.096 | 1.870 |
|  | Hypertension (%) | 191 (28.3) | 165 (33.1) | 0.072 | 1.258 | 0.979 | 1.617 |
|  | Dyslipidemia (%) | 378 (55.9) | 310 (62.2) | **0.029** | 1.300 | 1.026 | 1.647 |
|  | Glucose intolerance (%) | 180 (26.6) | 144 (28.9) | 0.386 | 1.121 | 0.866 | 1.451 |
|  | Obesity—body mass index ≥30 kg/m^2^ (%) | 3 (0.4) | 4 (0.8) | 0.429 | 1.816 | 0.405 | 8.153 |
| Donor preoperative systolic blood pressure (mmHg, SD) |  | 122.6 (14.3) | 124.2 (14.6) | 0.078 |  |  |  |
| Donor preoperative diastolic blood pressure (mmHg, SD) |  | 73.1 (11.0) | 74.9 (10.5) | **0.004** |  |  |  |
| Donor preoperative total cholesterol level (mg/dL, SD) |  | 210.7 (36.4) | 212.5 (36.8) | 0.491 |  |  |  |
| Donor preoperative triglyceride level (mg/dL, SD) |  | 136.8 (82.2) | 143.5 (92.4) | 0.417 |  |  |  |
| Donor preoperative low-density lipoprotein cholesterol level (mg/dL, SD) |  | 122.7 (30.5) | 124.4 (30.8) | 0.219 |  |  |  |
| Donor preoperative high-density lipoprotein cholesterol level (mg/dL, SD) |  | 63.0 (16.1) | 62.7 (16.9) | 0.876 |  |  |  |
| Donor preoperative fasting glucose level (mg/dL, SD) |  | 99.1 (12.7) | 99.4 (11.9) | 0.355 |  |  |  |
| 75-g oral glucose tolerance test results—blood glucose level at 2 h after glucose administration (mg/dL, SD) |  | 131.2 (35.4) | 132.7 (39.5) | 0.877 |  |  |  |
| Donor HbA1c level (%, SD) |  | 5.7 (0.4) | 5.8 (0.4) | 0.507 |  |  |  |
| Donor body mass index (kg/m^2^, SD) |  | 22.8 (2.8) | 22.7 (2.8) | 0.544 |  |  |  |
| Preoperative eGFR (mL/min/1.73 m^2^, SD) |  | 73.3 (12.7) | 73.5 (13.5) | 0.572 |  |  |  |
| Split kidney function on Tc-99m DTPA scintigraphy (%, SD) |  | 48.3 (3.8) | 47.8 (3.6) | **0.013** |  |  |  |
| Preoperative urine albumin/Cr ratio (mg/gCr, SD) |  | 10.2 (14.8) | 9.3 (9.5) | 0.897 |  |  |  |
| Baseline biopsy findings at 1 h after transplantation (%) |  | 357 (53.3) | 291 (59.3) | **0.043** | 1.276 | 1.008 | 1.614 |
| **RECIPIENT** |  |  |  |  |  |  |  |
| Recipient age (years, SD) |  | 48.5 (14.2) | 49.5 (13.2) | 0.299 |  |  |  |
| Recipient sex (male, %) |  | 437 (64.6) | 302 (60.6) | 0.161 | 0.843 | 0.663 | 1.070 |
| Recipient body mass index (kg/m^2^, SD) |  | 22.3 (3.8) | 22.6 (3.6) | 0.234 |  |  |  |
| Recipient follow-up period (months, SD) |  | 75.7 (45.1) | 76.1 (43.7) | 0.816 |  |  |  |
| Transplantation from first-degree relative donors (%) |  | 311 (46.0) | 229 (46.0) | 0.994 | 0.999 | 0.792 | 1.260 |
| Preoperative flow cytometry T cell crossmatch (positive, %) |  | 18 (2.7) | 23 (4.6) | 0.071 | 1.770 | 0.945 | 3.317 |
| Preoperative flow cytometry B cell crossmatch (positive, %) |  | 51 (7.5) | 63 (12.7) | **0.003** | 1.775 | 1.203 | 2.619 |
| Dialysis vintage (months, SD) |  | 61.8 (255.4) | 84.8 (493.1) | 0.996 |  |  |  |
| Preoperative ejection fraction on ultrasonographic cardiography (%) |  | 61.7 (7.8) | 62.1 (7.6) | 0.618 |  |  |  |
| Preoperative ventricular wall motion asynergy on ultrasonographic cardiography (%) |  | 80 (11.9) | 66 (13.3) | 0.464 | 1.139 | 0.804 | 1.614 |
| Preoperative sensitization—transfusion, pregnancy, transplantation (%) |  | 268 (39.6) | 212 (42.6) | 0.314 | 1.128 | 0.892 | 1.428 |
| HLA-AB mismatch (SD) |  | 2.5 (1.0) | 2.5 (0.9) | 0.776 |  |  |  |
| HLA-DR mismatch (SD) |  | 1.4 (0.6) | 1.4 (0.6) | 0.950 |  |  |  |
| Preoperative PRA class I (positive, ≥5%, %) |  | 86 (12.7) | 83 (16.7) | 0.057 | 1.372 | 0.990 | 1.902 |
| Preoperative PRA class II (positive, ≥5%, %) |  | 47 (7.0) | 44 (8.8) | 0.233 | 1.297 | 0.845 | 1.991 |
| Preformed DSA (%) |  | 38 (5.6) | 45 (9.0) | **0.024** | 1.668 | 1.065 | 2.611 |
| ABO incompatible transplantation (%) |  | 212 (31.4) | 180 (36.1) | 0.086 | 1.239 | 0.970 | 1.582 |
| Preoperative desensitization (preoperative rituximab administration or splenectomy, preoperative double filtration plasmapheresis, plasmapheresis, or IVIG, %) |  | 240 (35.5) | 208 (41.8) | **0.029** | 1.303 | 1.027 | 1.653 |
| Calcineurin inhibitor administration at kidney transplantation | TAC (%) | 128 (18.9) | 101 (20.3) | 0.630 |  |  |  |
|  | CsA (%) | 247 (36.5) | 189 (38.0) |  |  |  |  |
|  | TACER (%) | 301 (44.5) | 208 (41.8) |  |  |  |  |
| Calcineurin inhibitor administration at best eGFR within 3 weeks after kidney transplantation | TAC (%) | 128 (18.9) | 99 (19.9) | 0.759 |  |  |  |
|  | CsA (%) | 247 (36.5) | 188 (37.8) |  |  |  |  |
|  | TACER (%) | 301 (44.5) | 211 (42.4) |  |  |  |  |
| Calcineurin inhibitor administration at 1 week after kidney transplantation | TAC (%) | 128 (18.9) | 97 (19.5) | 0.630 |  |  |  |
|  | CsA (%) | 247 (36.5) | 193 (38.8) |  |  |  |  |
|  | TACER (%) | 301 (44.5) | 208 (41.8) |  |  |  |  |
| Calcineurin inhibitor administration at 2 weeks after kidney transplantation | TAC (%) | 127 (18.8) | 100 (20.1) | 0.582 |  |  |  |
|  | CsA (%) | 247 (36.6) | 191 (38.4) |  |  |  |  |
|  | TACER (%) | 301 (44.6) | 207 (41.6) |  |  |  |  |
| Calcineurin inhibitor administration at 3 weeks after kidney transplantation | TAC (%) | 120 (18.3) | 93 (19.1) | 0.533 |  |  |  |
|  | CsA (%) | 236 (35.9) | 187 (38.4) |  |  |  |  |
|  | TACER (%) | 301 (45.8) | 207 (42.5) |  |  |  |  |
| MMF, MZ, or EVR administration at transplantation | MMF (%) | 553 (81.8) | 391 (78.5) | 0.372 |  |  |  |
|  | MZ (%) | 19 (2.8) | 17 (3.4) |  |  |  |  |
|  | EVR (%) | 104 (15.4) | 90 (18.1) |  |  |  |  |
| Postoperative best eGFR within 3 weeks (mL/min/1.73 m^2^, SD) |  | 59.5 (16.8) | 55.2 (16.0) | **<0.001** |  |  |  |
| Postoperative eGFR at 1 week (mL/min/1.73 m^2^, SD) |  | 51.8 (15.3) | 47.3 (15.7) | **<0.001** |  |  |  |
| Postoperative eGFR at 2 weeks (mL/min/1.73 m^2^, SD) |  | 51.1 (14.6) | 46.7 (15.0) | **<0.001** |  |  |  |
| Postoperative eGFR at 3 weeks (mL/min/1.73 m^2^, SD) |  | 50.2 (14.6) | 46.4 (14.1) | **<0.001** |  |  |  |
| Trough levels of calcineurin inhibitor | TAC at best eGFR (ng/mL) | 11.3 (4.6) | 10.9 (5.0) | 0.541 |  |  |  |
|  | TAC at 1 week (ng/mL) | 11.4 (3.9) | 11.2 (3.8) | 0.929 |  |  |  |
|  | TAC at 2 weeks (ng/mL) | 10.7 (2.9) | 10.5 (3.2) | 0.566 |  |  |  |
|  | TAC at 3 weeks (ng/mL) | 9.9 (2.5) | 9.8 (2.9) | 0.578 |  |  |  |
|  | CsA at best eGFR (ng/mL) | 264.2 (112.7) | 263.8 (102.5) | 0.767 |  |  |  |
|  | CsA at 1 week (ng/mL) | 269.0 (102.1) | 275.6 (100.7) | 0.294 |  |  |  |
|  | CsA at 2 weeks (ng/mL) | 256.7 (97.1) | 257.9 (98.8) | 0.955 |  |  |  |
|  | CsA at 3 weeks (ng/mL) | 236.7 (90.6) | 244.5 (93.7) | 0.421 |  |  |  |
|  | TACER at best eGFR (ng/mL) | 7.4 (2.8) | 7.4 (2.6) | 0.635 |  |  |  |
|  | TACER at 1 week (ng/mL) | 7.9 (3.0) | 7.8 (3.2) | 0.414 |  |  |  |
|  | TACER at 2 weeks (ng/mL) | 7.6 (2.2) | 7.6 (2.4) | 0.890 |  |  |  |
|  | TACER at 3 weeks (ng/mL) | 7.5 (1.8) | 7.5 (2.2) | 0.558 |  |  |  |
| Conversion of immunosuppressive regimen within 1 month after kidney transplantation (%) |  | 0 | 15 (3.0) | **<0.001** |  |  |  |
| Recurrence of nephritis within 1 month after kidney transplantation (%) |  | 0 | 7 (1.6) | **0.002** |  |  |  |
| Calcineurin inhibitor toxicity diagnosed by protocol biopsy at 1 month after kidney transplantation (%) |  | 0 | 90 (20.9) | **<0.001** |  |  |  |
| Rejection (clinical and pathological, %) |  | 0 | 39 (7.8) | **<0.001** |  |  |  |
| Graft outcome | Functioning graft (%) | 621 (91.9) | 438 (88.0) | 0.078 |  |  |  |
|  | Death censored graft loss (%) | 34 (5.0) | 39 (7.8) |  |  |  |  |
|  | Death with functioning graft (%) | 21 (3.1) | 21 (4.2) |  |  |  |  |
| Graft survival period (months, SD) |  | 74.2 (44.8) | 73.7 (43.3) | 0.930 |  |  |  |
| Recipient death (%) |  | 27 (4.0) | 25 (5.0) | 0.397 | 1.271 | 0.728 | 2.218 |
| Abbreviations: CsA, cyclosporine A; DSA, donor-specific anti-human leukocyte antigen antibody; eGFR, estimated glomerular filtration rate; EVR, everolimus; HbA1c, hemoglobin A1c; HLA, human leukocyte antigen; IVIG, intravenous immunoglobulin; MMF, mycophenolate mofetil; MZ, mizoribine; PRA, panel reactive antibody; SD, standard deviation; TAC, tacrolimus; TACER, extended-release tacrolimus; Tc-99m DTPA, technetium-99m diethylene triamine pentaacetic acid. The bold font indicates statistically significant results. | | | | | | | |

| **Supplementary Table S5.** Donor and recipient operative outcomes | | | | | | | |
| --- | --- | --- | --- | --- | --- | --- | --- |
|  |  | Recipients for prediction models | Excluded recipients from prediction models | *P-*value | Odds ratio | 95% confidence interval | |
|  |  | n=676 | n=498 |  |  | Lower limit | Upper limit |
| **DONOR OPERATION** |  |  |  |  |  |  |  |
| Kidney laterality (left, %) |  | 620 (91.7) | 457 (91.8) | 0.975 | 1.007 | 0.661 | 1.533 |
| Kidney weight (g, SD) |  | 176.6 (40.7) | 179.2 (44.2) | 0.491 |  |  |  |
| Warm ischemia time (s, SD) |  | 133.6 (55.9) | 150.1 (82.4) | **<0.001** |  |  |  |
| Operating time (min, SD) |  | 207.7 (100.9) | 211.3 (78.8) | **0.022** |  |  |  |
| Operation blood loss (mL, SD) |  | 30.9 (36.9) | 40.9 (100.8) | 0.632 |  |  |  |
| Adverse events | Arterial injury (%) | 0 | 1 (0.2) | 0.424 |  |  |  |
|  | Venous injury (%) | 0 | 2 (0.4) | 0.099 |  |  |  |
|  | Open conversion (%) | 0 | 3 (0.6) | **0.043** |  |  |  |
|  | Intraoperative bleeding (%) | 0 | 2 (0.4) | 0.099 |  |  |  |
|  | Subcapsular hematoma (%) | 0 | 2 (0.4) | 0.099 |  |  |  |
|  | Bowel injury (%) | 0 | 1 (0.2) | 0.424 |  |  |  |
| Operation methods of donor nephrectomy | Hand-assisted laparoscopic (%) | 640 (94.7) | 469 (94.2) | **0.043** |  |  |  |
|  | Non-hand assisted retro-peritoneoscopic (%) | 29 (4.3) | 15 (3.0) |  |  |  |  |
|  | Open (%) | 7 (1.0) | 14 (2.8) |  |  |  |  |
| **RECIPIENT OPERATION** |  |  |  |  |  |  |  |
| Cold ischemia time (min, SD) |  | 87.4 (33.1) | 110.4 (44.6) | **<0.001** |  |  |  |
| Arterial reconstruction or ligation of thin upper pole artery (%) | | 0 | 336 (67.4) | **<0.001** |  |  |  |
| Recipient perioperative adverse events | Delayed graft function (%) | 0 | 1 (0.2) | 0.424 |  |  |  |
|  | Surgical site infection (%) | 0 | 15 (3.0) | **<0.001** |  |  |  |
|  | Arterial thrombosis (%) | 0 | 1 (0.2) | 0.424 |  |  |  |
|  | Arterial stenosis (%) | 0 | 4 (0.8) | **0.032** |  |  |  |
|  | Urine leakage (%) | 0 | 11 (2.2) | **<0.001** |  |  |  |
|  | Ureteral necrosis (%) | 0 | 2 (0.4) | 0.180 |  |  |  |
|  | Ureteral stenosis (%) | 0 | 4 (0.8) | **0.032** |  |  |  |
|  | Lymphocele (%) | 0 | 14 (2.8) | **<0.001** |  |  |  |
|  | Incisional hernia (%) | 0 | 5 (1.0) | **0.014** |  |  |  |
|  | Postoperative bleeding requiring reoperation (%) | 0 | 17 (3.4) | **<0.001** |  |  |  |
|  | Gastrointestinal bleeding or perforation (%) | 0 | 2 (0.4) | 0.180 | 0.423 | 0.396 | 0.452 |
|  | Colon perforation (%) | 0 | 3 (0.6) | 0.076 |  |  |  |
|  | Severe pneumonia (%) | 0 | 1 (0.2) | 0.424 |  |  |  |
| Abbreviation: SD, standard deviation. The bold font indicates statistically significant results. | | | | |  |  |  |

| **Supplementary Table S6.** Donor and recipient characteristics | | | | | | | |
| --- | --- | --- | --- | --- | --- | --- | --- |
|  |  | Recipients for the training set | Recipients for the validation set | *P-*value | Odds ratio | 95% confidence interval | |
|  |  | n=474 | n=202 |  |  | Lower limit | Upper limit |
| **DONOR** |  |  |  |  |  |  |  |
| Donor age (years, SD) |  | 58.8 (10.1) | 57.7 (9.9) | 0.190 |  |  |  |
| Donor sex (male, %) |  | 145 (30.6) | 78 (16.5) | **0.042** | 1.427 | 1.012 | 2.013 |
| Donation to first-degree relative recipients (%) |  | 223 (47.0) | 88 (18.6) | 0.406 | 0.869 | 0.624 | 1.210 |
| Smoking history (%) |  | 185 (39.0) | 92 (19.4) | 0.115 | 1.307 | 0.937 | 1.822 |
| Preoperative comorbidities ≥1 (%) |  | 335 (70.7) | 141 (69.8) | 0.820 | 0.959 | 0.669 | 1.374 |
|  | Hypertension (%) | 134 (28.3) | 57 (28.2) | 0.989 | 0.997 | 0.692 | 1.438 |
|  | Dyslipidemia (%) | 270 (57.0) | 108 (53.5) | 0.402 | 0.868 | 0.624 | 1.209 |
|  | Glucose intolerance (%) | 135 (28.5) | 45 (22.3) | 0.095 | 0.720 | 0.489 | 1.060 |
|  | Obesity—body mass index ≥30 kg/m^2^ (%) | 3 (0.6) | 0 | 0.558 |  |  |  |
| Donor preoperative systolic blood pressure (mmHg, SD) |  | 122.2 (14.1) | 123.4 (14.7) | 0.461 |  |  |  |
| Donor preoperative diastolic blood pressure (mmHg, SD) |  | 72.9 (10.9) | 73.6 (11.4) | 0.586 |  |  |  |
| Donor preoperative total cholesterol level (mg/dL, SD) |  | 211.0 (34.7) | 209.8 (40.3) | 0.576 |  |  |  |
| Donor preoperative triglyceride level (mg/dL, SD) |  | 137.6 (83.9) | 134.9 (78.4) | 0.772 |  |  |  |
| Donor preoperative low-density lipoprotein cholesterol level (mg/dL, SD) |  | 122.8 (30.4) | 122.5 (30.6) | 0.797 |  |  |  |
| Donor preoperative high-density lipoprotein cholesterol level (mg/dL, SD) |  | 63.3 (15.4) | 62.4 (17.6) | 0.898 |  |  |  |
| Donor preoperative fasting glucose level (mg/dL, SD) |  | 99.0 (13.2) | 99.3 (11.6) | 0.484 |  |  |  |
| 75-g oral glucose tolerance test results—blood glucose level at 2 h after glucose administration (mg/dL, SD) |  | 132.3 (35.8) | 128.7 (34.4) | 0.312 |  |  |  |
| Donor HbA1c level (%, SD) |  | 5.7 (0.3) | 5.7 (0.4) | 0.399 |  |  |  |
| Donor body mass index (kg/m^2^, SD) |  | 22.7 (2.8) | 23.2 (2.9) | 0.054 |  |  |  |
| Preoperative eGFR (mL/min/1.73 m^2^, SD) |  | 73.2 (12.9) | 73.5 (12.1) | 0.864 |  |  |  |
| Split kidney function on Tc-99m DTPA scintigraphy (%, SD) |  | 48.3 (3.7) | 48.3 (4.0) | 0.739 |  |  |  |
| Preoperative urine albumin/Cr ratio (mg/gCr, SD) |  | 10.3 (15.8) | 10.1 (12.0) | 0.855 |  |  |  |
| Baseline biopsy findings at 1 h after transplantation (%) |  | 253 (53.9) | 104 (51.7) | 0.600 | 0.915 | 0.657 | 1.274 |
| **RECIPIENT** |  |  |  |  |  |  |  |
| Recipient age (years, SD) |  | 48.4 (14.0) | 48.8 (14.7) | 0.584 |  |  |  |
| Recipient sex (male, %) |  | 315 (66.5) | 122 (60.4) | 0.131 | 0.770 | 0.548 | 1.082 |
| Recipient body mass index (kg/m^2^, SD) |  | 22.3 (3.6) | 22.4 (4.1) | 0.935 |  |  |  |
| Recipient follow-up period (months, SD) |  | 75.7 (44.9) | 75.8 (45.6) | 0.969 |  |  |  |
| Transplantation from first-degree relative donor (%) |  | 223 (47.0) | 88 (43.6) | 0.406 | 0.869 | 0.624 | 1.210 |
| Preoperative flow cytometry T cell crossmatch (positive, %) |  | 8 (1.7) | 10 (5.0) | **0.033** | 3.034 | 1.179 | 7.804 |
| Preoperative flow cytometry B cell crossmatch (positive, %) |  | 39 (8.2) | 12 (5.9) | 0.343 | 0.704 | 0.361 | 1.375 |
| Dialysis vintage (months, SD) |  | 59.6 (281.9) | 67.0 (179.1) | 0.282 |  |  |  |
| Preoperative ejection fraction on ultrasonographic cardiography (%) |  | 61.7 (7.6) | 61.7 (8.5) | 0.738 |  |  |  |
| Preoperative ventricular wall motion asynergy on ultrasonographic cardiography (%) |  | 57 (12.1) | 23 (11.4) | 0.807 | 0.938 | 0.560 | 1.569 |
| Preoperative sensitization—transfusion, pregnancy, transplantation (%) |  | 182 (38.4) | 86 (42.6) | 0.309 | 1.189 | 0.851 | 1.662 |
| HLA-AB mismatch (SD) |  | 2.5 (1.0) | 2.4 (1.0) | 0.322 |  |  |  |
| HLA-DR mismatch (SD) |  | 1.4 (0.6) | 1.4 (0.6) | 0.513 |  |  |  |
| Preoperative PRA class I (positive, ≥5%, %) |  | 54 (11.4) | 32 (15.8) | 0.112 | 1.464 | 0.913 | 2.348 |
| Preoperative PRA class II (positive, ≥5%, %) |  | 34 (7.2) | 13 (6.4) | 0.869 | 0.890 | 0.459 | 1.725 |
| Preformed DSA (%) |  | 22 (4.6) | 16 (7.9) | 0.101 | 1.767 | 0.908 | 3.441 |
| ABO-incompatible transplantation (%) |  | 155 (32.7) | 57 (28.2) | 0.250 | 0.809 | 0.564 | 1.161 |
| Preoperative desensitization (preoperative rituximab administration or splenectomy, preoperative double filtration plasmapheresis, plasmapheresis, or IVIG, %) |  | 172 (36.3) | 68 (33.7) | 0.514 | 0.891 | 0.630 | 1.260 |
| Calcineurin inhibitor administration at kidney transplantation | TAC (%) | 90 (19.0) | 38 (18.8) | 0.925 |  |  |  |
|  | CsA (%) | 171 (36.1) | 76 (37.6) |  |  |  |  |
|  | TACER (%) | 213 (44.9) | 88 (43.6) |  |  |  |  |
| MMF, MZ, or EVR administration at transplantation | MMF (%) | 384 (81.0) | 169 (83.7) | 0.634 |  |  |  |
|  | MZ (%) | 13 (2.7) | 6 (3.0) |  |  |  |  |
|  | EVR (%) | 77 (16.2) | 27 (13.4) |  |  |  |  |
| Postoperative best eGFR within 3 weeks (mL/min/1.73 m^2^, SD) |  | 59.0 (16.0) | 60.5 (18.7) | 0.615 |  |  |  |
| Postoperative eGFR at 1 week (mL/min/1.73 m^2^, SD) |  | 51.5 (14.7) | 52.3 (16.8) | 0.753 |  |  |  |
| Postoperative eGFR at 2 weeks (mL/min/1.73 m^2^, SD) |  | 50.7 (13.8) | 51.9 (16.3) | 0.772 |  |  |  |
| Postoperative eGFR at 3 weeks (mL/min/1.73 m^2^, SD) |  | 50.0 (14.0) | 50.6 (15.9) | 0.918 |  |  |  |
| Trough levels of calcineurin inhibitor | TAC at best eGFR (ng/mL) | 11.4 (4.9) | 11.2 (4.1) | 0.943 |  |  |  |
|  | TAC at 1 week (ng/mL) | 11.3 (4.2) | 11.6 (3.4) | 0.332 |  |  |  |
|  | TAC at 2 weeks (ng/mL) | 10.6 (2.9) | 10.7 (2.9) | 0.857 |  |  |  |
|  | TAC at 3 weeks (ng/mL) | 10.0 (2.6) | 9.9 (.2) | 0.782 |  |  |  |
|  | CsA at best eGFR (ng/mL) | 263.2 (112.6) | 266.5 (113.8) | 0.964 |  |  |  |
|  | CsA at 1 week (ng/mL) | 270.0 (101.5) | 266.7 (104.1) | 0.673 |  |  |  |
|  | CsA at 2 weeks (ng/mL) | 260.3 (98.9) | 248.4 (93.1) | 0.480 |  |  |  |
|  | CsA at 3 weeks (ng/mL) | 235.2 (89.3) | 240.2 (94.2) | 0.840 |  |  |  |
|  | TACER at best eGFR (ng/mL) | 7.5 (2.9) | 7.3 (2.6) | 0.891 |  |  |  |
|  | TACER at 1 week (ng/mL) | 7.8 (3.0) | 8.1 (3.0) | 0.197 |  |  |  |
|  | TACER at 2 weeks (ng/mL) | 7.5 (2.2) | 7.6 (2.2) | 0.890 |  |  |  |
|  | TACER at 3 weeks (ng/mL) | 7.5 (1.8) | 7.5 (1.8) | 0.707 |  |  |  |
| Conversion of immunosuppressive regimen within 1 month after kidney transplantation (%) |  | 0 | 0 | n.c. |  |  |  |
| Recurrence of nephritis within 1 month after kidney transplantation (%) |  | 0 | 0 | n.c. |  |  |  |
| Calcineurin inhibitor toxicity diagnosed by protocol biopsy at 1 month after kidney transplantation (%) |  | 0 | 0 | n.c. |  |  |  |
| Rejection (clinical and pathological, %) |  | 0 | 0 | n.c. |  |  |  |
| Graft outcome | Functioning graft (%) | 432 (91.1) | 189 (93.6) | 0.573 |  |  |  |
|  | Death censored graft loss (%) | 26 (5.5) | 8 (4.0) |  |  |  |  |
|  | Death with functioning graft (%) | 16 (3.4) | 5 (2.5) |  |  |  |  |
| Graft survival period (months, SD) |  | 74.0 (44.4) | 75.0 (45.6) | 0.860 |  |  |  |
| Recipient death (%) |  | 21 (4.4) | 6 (3.0) | 0.520 | 0.659 | 0.262 | 1.658 |
| Abbreviations: CsA, cyclosporine A; DSA, donor-specific anti-human leukocyte antigen antibody; eGFR, estimated glomerular filtration rate; EVR, everolimus; HbA1c, hemoglobin A1c; HLA, human leukocyte antigen; IVIG, intravenous immunoglobulin; n.c., not calculated; MMF, mycophenolate mofetil; MZ, mizoribine; PRA, panel reactive antibody; SD, standard deviation; TAC, tacrolimus; TACER, extended-release tacrolimus; Tc-99m DTPA, technetium-99m diethylene triamine pentaacetic acid. The bold font indicates statistically significant results. | | | | | | | |

| **Supplementary Table S7.** Donor and recipient operative outcomes | | | | | | | |
| --- | --- | --- | --- | --- | --- | --- | --- |
|  |  | Recipients for training set | Recipients for validation set | *P-*value | Odds ratio | 95% confidence interval | |
|  |  | n=474 | n=202 |  |  | Lower limit | Upper limit |
| **DONOR OPERATION** |  |  |  |  |  |  |  |
| Kidney laterality (left, %) |  | 440 (92.8) | 180 (89.1) | 0.108 | 0.632 | 0.360 | 1.111 |
| Kidney weight (g, SD) |  | 175.5 (41.0) | 179.4 (39.8) | 0.169 |  |  |  |
| Warm ischemia time (s, SD) |  | 134.6 (61.9) | 131.4 (38.2) | 0.302 |  |  |  |
| Operating time (min, SD) |  | 209.2 (115.8) | 204.1 (51.0) | 0.836 |  |  |  |
| Operation blood loss (mL, SD) |  | 30.6 (35.7) | 31.6 (39.6) | 0.952 |  |  |  |
| Adverse events | Arterial injury (%) | 0 | 0 | n.c. |  |  |  |
|  | Venous injury (%) | 0 | 0 | n.c. |  |  |  |
|  | Open conversion (%) | 0 | 0 | n.c. |  |  |  |
|  | Intraoperative bleeding (%) | 0 | 0 | n.c. |  |  |  |
|  | Subcapsular hematoma (%) | 0 | 0 | n.c. |  |  |  |
|  | Bowel injury (%) | 0 | 0 | n.c. |  |  |  |
| Operation methods of donor nephrectomy | Hand-assisted laparoscopic (%) | 448 (94.5) | 192 (95.0) | 0.599 |  |  |  |
|  | Non-hand assisted retro-peritoneoscopic (%) | 22 (4.6) | 7 (3.5) |  |  |  |  |
|  | Open (%) | 4 (0.8) | 3 (1.5) |  |  |  |  |
| **RECIPIENT OPERATION** |  |  |  |  |  |  |  |
| Cold ischemia time (min, SD) |  | 89.4 (35.6) | 82.7 (26.1) | 0.131 |  |  |  |
| Arterial reconstruction or ligation of thin upper pole artery (%) | | 0 | 0 | n.c. |  |  |  |
| Recipient perioperative adverse events | Delayed graft function (%) | 0 | 0 | n.c. |  |  |  |
|  | Surgical site infection (%) | 0 | 0 | n.c. |  |  |  |
|  | Arterial thrombosis (%) | 0 | 0 | n.c. |  |  |  |
|  | Arterial stenosis (%) | 0 | 0 | n.c. |  |  |  |
|  | Urine leakage (%) | 0 | 0 | n.c. |  |  |  |
|  | Ureteral necrosis (%) | 0 | 0 | n.c. |  |  |  |
|  | Ureteral stenosis (%) | 0 | 0 | n.c. |  |  |  |
|  | Lymphocele (%) | 0 | 0 | n.c. |  |  |  |
|  | Incisional hernia (%) | 0 | 0 | n.c. |  |  |  |
|  | Postoperative bleeding requiring reoperation (%) | 0 | 0 | n.c. |  |  |  |
|  | Gastrointestinal bleeding or perforation (%) | 0 | 0 | n.c. |  |  |  |
|  | Colon perforation (%) | 0 | 0 | n.c. |  |  |  |
|  | Severe pneumonia (%) | 0 | 0 | n.c. |  |  |  |
| Abbreviation: n.c., not calculated; SD, standard deviation. The bold font indicates statistically significant results. | | | | | | | |

| **Supplementary Table S8.** Results of 10-fold cross-validation for ideal best eGFR within 3 weeks after kidney transplantation | | |
| --- | --- | --- |
| Model | R | R-squared |
| 1 | 0.521 | 0.272 |
| 2 | 0.600 | 0.360 |
| 3 | 0.627 | 0.394 |
| **4** | **0.646** | **0.418** |
| 5 | 0.598 | 0.358 |
| 6 | 0.284 | 0.080 |
| 7 | 0.589 | 0.347 |
| 8 | 0.533 | 0.284 |
| 9 | 0.621 | 0.386 |
| 10 | 0.598 | 0.358 |
| Abbreviations: eGFR, estimated glomerular filtration rate. The bold font indicates the best model. | | |

| **Supplementary Table S9.** The best prediction model for ideal best eGFR within 3 weeks after kidney transplantation | | | | | | |
| --- | --- | --- | --- | --- | --- | --- |
| Model | Variables | Partial regression coefficient | Standard deviation | Standardized partial regression coefficient | t-value | *P-*value |
| 4 | Intercept | 51.411 | 10.425 |  | 4.932 | <0.001 |
|  | Recipient body mass index (kg/m^2^) | -1.797 | 0.154 | -0.402 | -11.676 | <0.001 |
|  | Donor age (years) | -0.364 | 0.064 | -0.213 | -5.682 | <0.001 |
|  | Donor preoperative eGFR (mL/min/1.73 m^2^) | 0.362 | 0.049 | 0.262 | 7.353 | <0.001 |
|  | Donor body mass index (kg/m^2^) | 0.963 | 0.207 | 0.161 | 4.647 | <0.001 |
|  | Male donor (vs. female) | 4.921 | 1.228 | 0.136 | 4.008 | <0.001 |
|  | Warm ischemia time (s) | 0.036 | 0.010 | 0.121 | 3.663 | <0.001 |
|  | Donor split kidney function of grafted kidney on Tc-99m DTPA scintigraphy (%) | 0.328 | 0.134 | 0.082 | 2.448 | 0.015 |
|  | Calcineurin inhibitor (extended release tacrolimus vs. other calcineurin inhibitors) | 2.842 | 1.132 | 0.082 | 2.510 | 0.012 |
|  | Open nephrectomy (vs. endoscopic nephrectomy) | 19.038 | 6.017 | 0.111 | 3.164 | 0.002 |
|  | Blood loss at donor nephrectomy (mL) | -0.044 | 0.017 | -0.091 | -2.546 | 0.011 |
|  | Pathological findings at 1 h after kidney transplantation (vs. non-pathological findings at 1 h after kidney transplantation) | -2.432 | 1.216 | -0.071 | -2.000 | 0.046 |
| Abbreviation: eGFR, estimated glomerular filtration rate; Tc-99m DTPA, technetium-99m diethylene triamine pentaacetic acid. | | | | | | |

| **Supplementary Table S10.** Results of the 10-fold cross-validation for ideal eGFR at 1 week after kidney transplantation | | |
| --- | --- | --- |
| Model | R | R-squared |
| 1 | 0.416 | 0.173 |
| 2 | 0.545 | 0.297 |
| 3 | 0.463 | 0.215 |
| 4 | 0.562 | 0.316 |
| 5 | 0.558 | 0.311 |
| 6 | 0.273 | 0.075 |
| **7** | **0.573** | **0.328** |
| 8 | 0.520 | 0.271 |
| 9 | 0.424 | 0.179 |
| 10 | 0.513 | 0.263 |
| Abbreviations: eGFR, estimated glomerular filtration rate. The bold font indicates the best model. | | |

| **Supplementary Table S11.** The best prediction model for ideal eGFR at 1 week after kidney transplantation | | | | | | |
| --- | --- | --- | --- | --- | --- | --- |
| Model | Variables | Partial regression coefficient | Standard deviation | Standardized partial regression coefficient | t-value | *P*-value |
| 7 | Intercept | 65.198 | 7.815 |  | 8.342 | <0.001 |
|  | Recipient body mass index (kg/m^2^) | -1.458 | 0.154 | -0.355 | -9.468 | <0.001 |
|  | Donor age (years) | -0.256 | 0.062 | -0.167 | -4.138 | <0.001 |
|  | Donor preoperative eGFR (mL/min/1.73 m^2^) | 0.285 | 0.048 | 0.221 | 5.922 | <0.001 |
|  | Donor body mass index (kg/m^2^) | 0.713 | 0.195 | 0.130 | 3.647 | <0.001 |
|  | Calcineurin inhibitor (extended release tacrolimus vs. other calcineurin inhibitors) | 3.773 | 1.088 | 0.121 | 3.468 | 0.001 |
|  | Pathological findings at 1 h after kidney transplantation (vs. non-pathological findings at 1 h after kidney transplantation) | -3.158 | 1.163 | -0.102 | -2.716 | 0.007 |
|  | Recipient age (years) | -0.093 | 0.039 | -0.085 | -2.377 | 0.018 |
|  | Open nephrectomy (vs. endoscopic nephrectomy) | 13.417 | 4.999 | 0.093 | 2.684 | 0.007 |
|  | Warm ischemia time (s) | 0.021 | 0.009 | 0.077 | 2.227 | 0.026 |
|  | Recipient male (vs. female) | -2.345 | 1.187 | -0.072 | -1.975 | 0.049 |
| Abbreviation: eGFR, estimated glomerular filtration rate. | | | | | | |

| **Supplementary Table S12.** Results of the 10-fold cross-validation for ideal eGFR at 2 weeks after kidney transplantation | | |
| --- | --- | --- |
| Model | R | R-squared |
| 1 | 0.425 | 0.181 |
| 2 | 0.556 | 0.309 |
| 3 | 0.473 | 0.224 |
| 4 | 0.610 | 0.373 |
| 5 | 0.529 | 0.280 |
| 6 | 0.253 | 0.064 |
| **7** | **0.619** | **0.383** |
| 8 | 0.514 | 0.264 |
| 9 | 0.583 | 0.339 |
| 10 | 0.599 | 0.359 |
| Abbreviations: eGFR, estimated glomerular filtration rate. The bold font indicates the best model. | | |

| **Supplementary Table S13.** The best prediction model for ideal eGFR at 2 weeks after kidney transplantation | | | | | | |
| --- | --- | --- | --- | --- | --- | --- |
| Model | Variables | Partial regression coefficient | Standard deviation | Standardized partial regression coefficient | t-value | *P*-value |
| 7 | Intercept | 59.081 | 7.210 |  | 8.194 | <0.001 |
|  | Recipient body mass index (kg/m^2^) | -1.316 | 0.140 | -0.335 | -9.400 | <0.001 |
|  | Donor age (years) | -0.308 | 0.057 | -0.209 | -5.362 | <0.001 |
|  | Donor preoperative eGFR (mL/min/1.73 m^2^) | 0.286 | 0.045 | 0.231 | 6.412 | <0.001 |
|  | Warm ischemia time (s) | 0.038 | 0.009 | 0.150 | 4.465 | <0.001 |
|  | Donor body mass index (kg/m^2^) | 0.657 | 0.183 | 0.125 | 3.592 | <0.001 |
|  | Calcineurin inhibitor (extended release tacrolimus vs. other calcineurin inhibitors) | 3.841 | 1.002 | 0.129 | 3.832 | <0.001 |
|  | Sensitization by transplantation, pregnancy, and transfusion (non-sensitization) | 2.877 | 1.087 | 0.095 | 2.647 | 0.008 |
|  | Open nephrectomy (vs. endoscopic nephrectomy) | 12.795 | 4.584 | 0.093 | 2.792 | 0.005 |
|  | Recipient age (years) | -0.087 | 0.037 | -0.083 | -2.376 | 0.018 |
|  | Pathological findings at 1 h after kidney transplantation (vs. non-pathological findings at 1 h after kidney transplantation) | -2.409 | 1.077 | -0.081 | -2.237 | 0.026 |
|  | Male donor (vs. female) | 2.347 | 1.132 | 0.075 | 2.073 | 0.039 |
| Abbreviation: eGFR, estimated glomerular filtration rate. | | | | | | |

| **Supplementary Table S14.** Results of the 10-fold cross-validation for ideal eGFR at 3 weeks after kidney transplantation | | |
| --- | --- | --- |
| Model | R | R-squared |
| 1 | 0.609 | 0.370 |
| 2 | 0.438 | 0.192 |
| 3 | 0.402 | 0.161 |
| 4 | 0.255 | 0.065 |
| 5 | 0.607 | 0.368 |
| 6 | 0.559 | 0.312 |
| **7** | **0.693** | **0.480** |
| 8 | 0.533 | 0.284 |
| 9 | 0.433 | 0.187 |
| 10 | 0.483 | 0.233 |
| Abbreviations: eGFR, estimated glomerular filtration rate. The bold font indicates the best model. | | |

| **Supplementary Table S15.** The best prediction model for ideal eGFR at 3 weeks after kidney transplantation | | | | | | |
| --- | --- | --- | --- | --- | --- | --- |
| Model | Variables | Partial regression coefficient | Standard deviation | Standardized partial regression coefficient | t-value | *P*-value |
| 7 | Intercept | 56.605 | 7.527 |  | 7.521 | <0.001 |
|  | Donor age (years) | -0.285 | 0.059 | -0.192 | -4.798 | <0.001 |
|  | Recipient body mass index (kg/m^2^) | -1.173 | 0.142 | -0.299 | -8.246 | <0.001 |
|  | Donor preoperative eGFR (mL/min/1.73 m^2^) | 0.271 | 0.046 | 0.220 | 5.923 | <0.001 |
|  | Warm ischemia time (s) | 0.038 | 0.009 | 0.150 | 4.262 | <0.001 |
|  | Male donor (vs. female) | 4.066 | 1.118 | 0.132 | 3.638 | <0.001 |
|  | Calcineurin inhibitor (extended release tacrolimus vs. other calcineurin inhibitors) | 12.204 | 3.269 | 0.414 | 3.733 | <0.001 |
|  | Donor body mass index (kg/m^2^) | 0.662 | 0.191 | 0.125 | 3.457 | 0.001 |
|  | Recipient trough levels of extended release tacrolimus (ng/mL) | -1.160 | 0.410 | -0.314 | -2.830 | 0.005 |
|  | Pathological findings at 1 h after kidney transplantation (vs. non-pathological findings at 1 h after kidney transplantation) | -3.003 | 1.101 | -0.102 | -2.726 | 0.007 |
|  | Open nephrectomy (vs. endoscopic nephrectomy) | 14.569 | 4.902 | 0.111 | 2.972 | 0.003 |
|  | Recipient age (years) | -0.084 | 0.037 | -0.082 | -2.253 | 0.025 |
|  | Blood loss at donor nephrectomy (mL) | -0.029 | 0.015 | -0.074 | -1.971 | 0.049 |
| Abbreviation: eGFR, estimated glomerular filtration rate. | | | | | | |

| **Supplementary Table S16.** Univariate Fine–Gray competing model analysis for graft loss | | | | | |
| --- | --- | --- | --- | --- | --- |
|  |  | Univariate analysis | | | |
|  |  | *P*-value | Hazard ratio | 95% confidence interval | |
|  |  |  |  | Lower limit | Upper limit |
| **Recipient characteristics** |  |  |  |  |  |
| Male recipient (vs. female) |  | **0.032** | 1.754 | 1.050 | 2.932 |
| Recipient age (years) |  | 0.470 | 0.993 | 0.976 | 1.011 |
| Recipient body mass index (kg/m^2^) |  | 0.150 | 1.042 | 0.985 | 1.103 |
| Transplantation from a first-degree relative donor (vs. non-transplantation from a first-degree relative donor) |  | 0.080 | 1.514 | 0.952 | 2.406 |
| Preoperative flow cytometry T cell cross match positive (vs. negative) |  | 0.770 | 1.232 | 0.299 | 5.070 |
| Preoperative flow cytometry B cell cross match positive (vs. negative) |  | 0.063 | 1.749 | 0.971 | 3.152 |
| Dialysis vintage (months) |  | 0.530 | 0.999 | 0.997 | 1.002 |
| Preoperative sensitization (transfusion, pregnancy, transplantation) (vs. non-preoperative sensitization) |  | 0.140 | 0.698 | 0.431 | 1.130 |
| HLA-AB mismatch |  | 0.440 | 0.917 | 0.736 | 1.143 |
| HLA-DR mismatch |  | 0.520 | 0.889 | 0.620 | 1.274 |
| Preoperative PRA class I positive (≥5%) (vs. negative) |  | 0.330 | 1.356 | 0.733 | 2.510 |
| Preoperative PRA class II positive (≥5%) (vs. negative) |  | 0.860 | 0.914 | 0.333 | 2.506 |
| Preformed DSA (vs. non-preformed DSA) |  | **0.013** | 2.260 | 1.185 | 4.311 |
| ABO-incompatible transplantation (vs non-ABO-incompatible transplantation) |  | 0.300 | 1.284 | 0.801 | 2.060 |
| Preoperative desensitization (preoperative rituximab administration or splenectomy, preoperative double filtration plasmapheresis, plasmapheresis, or IVIG) (vs. non-preoperative desensitization) |  | **0.028** | 1.670 | 1.056 | 2.639 |
| Calcineurin inhibitor administration at transplantation |  | 0.304 | (for all categories) |  |  |
|  | TAC | ref | 1.000 |  |  |
|  | CsA | 0.790 | 0.933 | 0.556 | 1.565 |
|  | TACER | 0.140 | 0.534 | 0.232 | 1.229 |
| MMF, MZ, or EVR administration at transplantation |  | 0.083 | (for all categories) |  |  |
|  | MMF | ref | 1.000 |  |  |
|  | MZ | 0.170 | 0.396 | 0.105 | 1.494 |
|  | EVR | 0.070 | 0.341 | 0.106 | 1.090 |
| Preoperative ejection fraction on ultrasonographic cardiography (%) |  | 0.690 | 0.993 | 0.962 | 1.026 |
| Preoperative ventricular wall motion asynergy on ultrasonographic cardiography (vs. non-preoperative ventricular wall motion asynergy on ultrasonographic cardiography) |  | 0.230 | 1.479 | 0.777 | 2.815 |
| Actual best eGFR within 3 weeks after transplantation (mL/min/1.73 m^2^) |  | 0.440 | 0.991 | 0.970 | 1.014 |
| Actual eGFR at 1 week after transplantation (mL/min/1.73 m^2^) |  | 0.500 | 0.992 | 0.971 | 1.015 |
| Actual eGFR at 2 weeks after transplantation (mL/min/1.73 m^2^) |  | 0.500 | 0.992 | 0.968 | 1.016 |
| Actual eGFR at 3 weeks after transplantation (mL/min/1.73 m^2^) |  | 0.280 | 0.984 | 0.956 | 1.013 |
| Predicted ideal best eGFR within 3 weeks after transplantation (mL/min/1.73 m^2^) |  | 0.330 | 0.989 | 0.967 | 1.011 |
| Predicted ideal eGFR at 1 week after transplantation (mL/min/1.73 m^2^) |  | 0.200 | 0.980 | 0.951 | 1.010 |
| Predicted ideal eGFR at 2 weeks after transplantation (mL/min/1.73 m^2^) |  | 0.200 | 0.981 | 0.953 | 1.010 |
| Predicted ideal eGFR at 3 weeks after transplantation (mL/min/1.73 m^2^) |  | 0.190 | 0.989 | 0.973 | 1.005 |
| Predicted ideal best eGFR / actual best eGFR within 3 weeks after transplantation |  | **<0.001** | 1.823 | 1.497 | 2.220 |
| Predicted ideal eGFR / actual eGFR at 1 week after transplantation |  | **0.045** | 1.378 | 1.007 | 1.884 |
| Predicted ideal eGFR / actual eGFR at 2 weeks after transplantation |  | **0.008** | 1.407 | 1.094 | 1.809 |
| Predicted ideal eGFR / actual eGFR at 3 weeks after transplantation |  | **<0.001** | 1.706 | 1.491 | 1.951 |
| **Donor characteristics** |  |  |  |  |  |
| Male donor (vs. female) |  | >0.999 | 1.001 | 0.623 | 1.608 |
| Donor age (years) |  | **0.048** | 1.027 | 1.000 | 1.055 |
| Donor body mass index (kg/m^2^) |  | 0.370 | 1.038 | 0.957 | 1.126 |
| Baseline biopsy findings at 1 h after transplantation (vs. non-baseline biopsy findings 1 h after transplantation) |  | 0.910 | 1.027 | 0.645 | 1.634 |
| Preoperative comorbidities ≥1 (vs. non-preoperative comorbidity) |  | 0.090 | 1.683 | 0.922 | 3.073 |
| Smoking history (vs. non-smoking history) |  | 0.490 | 1.108 | 0.830 | 1.479 |
| Preoperative eGFR (mL/min/1.73 m^2^) |  | 0.620 | 0.996 | 0.980 | 1.012 |
| **Operation factors** |  |  |  |  |  |
| Kidney laterality (left) (vs. right) |  | 0.900 | 1.056 | 0.447 | 2.492 |
| Kidney weight (g) |  | 0.060 | 1.004 | 1.000 | 1.009 |
| Warm ischemia time (s) |  | 0.810 | 1.000 | 0.997 | 1.003 |
| Donor operation time (min) |  | 0.280 | 0.998 | 0.994 | 1.002 |
| Donor operation blood loss (mL) |  | 0.610 | 1.000 | 0.999 | 1.002 |
| Arterial reconstruction or ligation of thin upper pole artery (vs. non-arterial reconstruction or ligation of thin upper pole artery) |  | 0.920 | 0.973 | 0.582 | 1.626 |
| Split kidney function on scintigraphy (%) |  | 0.150 | 1.043 | 0.985 | 1.105 |
| Operation methods of donor nephrectomy |  | 0.093 | (for all categories) |  |  |
|  | Hand-assisted laparoscopic | ref | 1.000 |  |  |
|  | Non-hand assisted retro-peritoneoscopic | 0.200 | 0.510 | 0.184 | 1.416 |
|  | Open | 0.110 | 2.194 | 0.848 | 5.676 |
| Cold ischemia time (min) |  | 0.450 | 1.002 | 0.997 | 1.006 |
| Abbreviations: CsA, cyclosporin A; DSA, donor-specific anti-human leukocyte antigen antibody; eGFR, estimated glomerular filtration rate; EVR, everolimus; HLA, human leukocyte antigen; IVIG, intravenous immunoglobulin; MMF, mycophenolate mofetil; MZ, mizoribine; PRA, panel reactive antibody; ref, reference; TAC, tacrolimus; TACER, extended-release tacrolimus; Tc-99m DTPA, technetium-99m diethylene triamine pentaacetic acid. The bold font indicates statistically significant results. | | | | | |

| **Supplementary Table S17 (A).** Multivariate Fine–Gray competing model analysis for graft loss using predicted ideal best eGFR/actual best eGFR within 3 weeks after transplantation | | | | |
| --- | --- | --- | --- | --- |
|  | *P*-value | Hazard ratio | 95% confidence interval | |
|  |  |  | Lower limit | Upper limit |
| Predicted ideal best eGFR/actual best eGFR within 3 weeks after transplantation | **<0.001** | 1.496 | 1.225 | 1.826 |
| Male recipient (vs. female) | **0.005** | 2.231 | 1.276 | 3.900 |
| Preformed DSA (vs. non-preformed DSA) | **0.016** | 2.446 | 1.182 | 5.061 |
| Preoperative desensitization (preoperative rituximab administration or splenectomy, preoperative double filtration plasmapheresis, plasmapheresis, or IVIG) (vs. non-preoperative desensitization) | 0.091 | 1.506 | 0.937 | 2.419 |
| Donor age (years) | 0.051 | 1.027 | 1.000 | 1.056 |
| Abbreviations: DSA, donor-specific anti-human leukocyte antigen antibody; eGFR, estimated glomerular filtration rate; IVIG, intravenous immunoglobulin. The bold font indicates statistically significant results. | | | | |

| **Supplementary Table S17 (B).** Multivariate Fine–Gray competing model analysis for graft loss using predicted ideal eGFR/actual eGFR at 1 week after transplantation | | | | |
| --- | --- | --- | --- | --- |
|  | *P*-value | Hazard ratio | 95% confidence interval | |
|  |  |  | Lower limit | Upper limit |
| Predicted ideal eGFR/actual eGFR at 1 week after transplantation | **0.006** | 1.309 | 1.079 | 1.588 |
| Male recipient (vs. female) | **0.003** | 2.338 | 1.346 | 4.062 |
| Preformed DSA (vs. non-preformed DSA) | **0.006** | 2.656 | 1.325 | 5.321 |
| Preoperative desensitization (preoperative rituximab administration or splenectomy, preoperative double filtration plasmapheresis, plasmapheresis, or IVIG) (vs. non-preoperative desensitization) | 0.091 | 1.505 | 0.937 | 2.416 |
| Donor age (years) | **0.045** | 1.028 | 1.001 | 1.057 |
| Abbreviations: DSA, donor-specific anti-human leukocyte antigen antibody; eGFR, estimated glomerular filtration rate; IVIG, intravenous immunoglobulin. The bold font indicates statistically significant results. | | | | |

| **Supplementary Table S17 (C).** Multivariate Fine–Gray competing model analysis for graft loss using predicted ideal eGFR/actual eGFR at 2 weeks after transplantation | | | | |
| --- | --- | --- | --- | --- |
|  | *P*-value | Hazard ratio | 95% confidence interval | |
|  |  |  | Lower limit | Upper limit |
| Predicted ideal eGFR/actual eGFR at 2 weeks after transplantation | **0.002** | 1.323 | 1.105 | 1.584 |
| Male recipient (vs. female) | **0.003** | 2.338 | 1.346 | 4.063 |
| Preformed DSA (vs. non-preformed DSA) | **0.006** | 2.670 | 1.336 | 5.337 |
| Preoperative desensitization (preoperative rituximab administration or splenectomy, preoperative double filtration plasmapheresis, plasmapheresis, or IVIG) (vs. non-preoperative desensitization) | 0.120 | 1.459 | 0.906 | 2.350 |
| Donor age (years) | **0.047** | 1.028 | 1.000 | 1.057 |
| Abbreviations: DSA, donor-specific anti-human leukocyte antigen antibody; eGFR, estimated glomerular filtration rate; IVIG, intravenous immunoglobulin. The bold font indicates statistically significant results. | | | | |

| **Supplementary Table S17 (D).** Multivariate Fine–Gray competing model analysis for graft loss using predicted ideal eGFR/actual eGFR at 3 weeks after transplantation | | | | |
| --- | --- | --- | --- | --- |
|  | *P*-value | Hazard ratio | 95% confidence interval | |
|  |  |  | Lower limit | Upper limit |
| Predicted ideal eGFR/actual eGFR at 3 weeks after transplantation | **<0.001** | 1.452 | 1.240 | 1.699 |
| Male recipient (vs. female) | **0.030** | 1.032 | 1.003 | 1.062 |
| Preformed DSA (vs. non-preformed DSA) | **0.023** | 2.518 | 1.136 | 5.584 |
| Preoperative desensitization (preoperative rituximab administration or splenectomy, preoperative double filtration plasmapheresis, plasmapheresis, or IVIG) (vs. non-preoperative desensitization) | 0.150 | 1.451 | 0.874 | 2.409 |
| Donor age (years) | **0.012** | 2.157 | 1.183 | 3.932 |
| Abbreviations: DSA, donor-specific anti-human leukocyte antigen antibody; eGFR, estimated glomerular filtration rate; IVIG, intravenous immunoglobulin. The bold font indicates statistically significant results. | | | | |
